# Cross-Species Triage of SERT-Oriented Polyheteroaryl Candidates Prioritizes AD20/UtIA-0108 as an Early Neuroactive Candidate

**DOI:** 10.3390/cimb48090922

**Published:** 2026-09-09

**Authors:** Maria V. Komelkova, Stanislav A. Fedorov, Veronika A. Isaeva, Irina A. Utepova, Yulia D. Grygoreva, Inessa Yu Bahareva, Vitalii S. Moskaliuk, Elena M. Kondaurova, Alexandr V. Zhdanov, Pavel O. Platkovskii, Maria A. Trestsova, Daria A. Andreeva, Maxim A. Perfilev, Andrey N. Kochetkov, Pavel M. Vassiliev, Vladimir S. Naumenko, Alexey P. Sarapultsev

**Affiliations:** 1 Russian-Chinese Education and Research Center of System Pathology, South Ural State University, 76 Lenin Prospekt, 454080 Chelyabinsk, Russia; mkomelkova@mail.ru (M.V.K.); irina-utepova@yandex.ru (I.A.U.);; 2 Laboratory of Immunopathophysiology, Institute of Immunology and Physiology, Ural Branch of the Russian Academy of Sciences, 106 Pervomaiskaya Street, 620049 Ekaterinburg, Russia; 3 Federal Research Center Institute of Cytology and Genetics, Siberian Branch of the Russian Academy of Sciences, 10 Prospekt Akademika Lavrent’yeva, 630090 Novosibirsk, Russia; 4 Department of Organic and Biomolecular Chemistry, Ural Federal University, 28 Mira Street, 620002 Ekaterinburg, Russia; 5 Institute of Organic Synthesis, Ural Branch of the Russian Academy of Sciences, 22 S. Kovalevskoy Street, 620066 Ekaterinburg, Russia; 6 Scientific Center for Innovative Medicines, Volgograd State Medical University, 1 Pavshikh Bortsov Sq., 400066 Volgograd, Russia

**Keywords:** AD20/UtIA-0108, zebrafish, drug discovery, molecular docking, SERT-oriented screening, chronic unpredictable stress, light–dark test, imidazo[1,2-a]pyridine, oxadiazolopyrazine, monoaminergic multitarget candidate

## Abstract

Depression and stress-related disorders remain important targets for the development of new neuroactive compounds. We evaluated three imidazo[1,2-a]pyridine–[1,2,5]oxadiazolo[3,4-b]pyrazine derivatives, selected by a SERT-oriented computational screen, using chemical characterization, zebrafish embryo toxicity, larval and adult zebrafish behavioural assays, mouse behavioural HPLC, and qPCR experiments. AD19/UtIA-0167 had the highest computational consensus but showed greater embryo toxicity and no consistent advantage in adult zebrafish. AD20/UtIA-0108 showed a distinct larval locomotor profile, increased middle-zone occupancy after chronic exposure in stressed adult zebrafish, and was therefore examined in mice. Chronic AD20 increased latency to enter the dark compartment; whereas, time in the light compartment, open-field measures and forced-swim immobility were unchanged. HPLC and qPCR changes were region-specific and did not demonstrate direct SERT engagement. AD20 should therefore be regarded as an early neuroactive candidate from a SERT-oriented screen, not as a confirmed SERT inhibitor or antidepressant. Interpretation across species is limited by the duplicated zebrafish serotonin-transporter system, waterborne exposure and the limited scope of the mouse behavioural effect. Direct transporter assays, pharmacokinetic studies and independent behavioural replication are required.

## 1. Introduction

Depressive and stress-related disorders remain among the most consequential targets for neuropharmacological research. According to recent World Health Organization estimates, depression affects hundreds of millions of people worldwide and is associated with substantial disability, suicide risk, impaired social functioning, and major economic costs [1,2]. Although effective treatments exist, access to treatment and treatment response remain incomplete; many patients do not receive adequate treatment, and a clinically important proportion of patients show an insufficient response to available pharmacological strategies [1,2,3]. Treatment-resistant depression is now recognized not as a marginal clinical subgroup but as a major public-health and therapeutic problem, complicated by heterogeneous definitions, biological diversity, and variable response to currently available interventions [3,4]. These considerations continue to support the search for new neuroactive small molecules and for more flexible preclinical screening pipelines.

The current antidepressant and anxiolytic drug-discovery landscape is no longer adequately represented by a single-target monoaminergic framework. Serotonergic, noradrenergic, dopaminergic, glutamatergic, GABAergic, neurotrophic, inflammatory, endocrine, and stress-regulatory mechanisms are increasingly considered as interacting rather than isolated therapeutic domains [4,5]. This does not reduce the importance of monoamine-related targets, including the serotonin transporter and serotonin receptors, but it changes the evidentiary standard for new compounds. A computationally favourable interaction with a monoaminergic target should be treated as a hypothesis-generating signal rather than as proof of mechanism. For early-stage candidates, especially those emerging from chemically diverse heterocyclic series, the central question is whether an in silico profile is accompanied by acceptable toxicity and coherent biological activity across independent behavioural and molecular assays.

Computational screening, molecular docking, and machine learning-based prioritization have become integral components of modern drug discovery. These approaches can reduce the experimental search space, identify plausible ligand–target interactions, prioritize compounds for synthesis or testing, and support mechanistic hypotheses before extensive in vivo work is undertaken [6,7]. At the same time, recent analyses emphasize that docking and deep learning models remain conditional tools. Their outputs depend on receptor structure, ligand state, scoring functions, protein flexibility, dataset bias, and the ability of models to generalize to new chemical scaffolds or binding pockets [7,8]. Therefore, computational ranking alone cannot establish pharmacological identity. A robust discovery design should combine in silico prioritization with staged biological validation, including toxicity gates, whole-organism phenotyping, and mammalian follow-up.

Zebrafish are useful for linking computational prioritization with mammalian testing. Larvae permit rapid behavioural screening in multiwell plates; whereas adult fish can be used in more complex stress-related paradigms [9,10,11]. The serotonin system is sufficiently conserved to make zebrafish relevant to monoaminergic drug discovery, but important species differences remain. Zebrafish possess two serotonin-transporter paralogues, *slc6a4a/serta* and *slc6a4b/sertb*, and their pharmacology is not identical to that of mammalian SERT [12,13,14]. Consequently, a behavioural effect in zebrafish cannot be assumed to reflect the same transporter interaction in mice. Interpretation also depends on developmental stage, exposure route, locomotor state and the behavioural paradigm used [10,11]. These considerations are particularly important for light–dark assays and chronic-stress models, in which changes in activity may have several explanations [15]. Recent reviews of zebrafish screening models likewise emphasize that experimental context and the selected endpoints determine how the findings should be interpreted [16].

The present study examined three imidazo[1,2-a]pyridine–[1,2,5]oxadiazolo[3,4-b]pyrazine derivatives identified using a SERT-oriented computational multitarget prioritization approach. AD19/UtIA-0167 and AD20/UtIA-0108 were compared in zebrafish, and AD20 was then evaluated in mice. Chemical synthesis and characterization, embryo toxicity, larval light–dark locomotion, adult novel-tank behaviour after chronic unpredictable stress, mouse behaviour, HPLC and qPCR were combined in the experimental program. Because direct SERT binding or uptake was not measured, the computational result was used only to select compounds for biological testing.

The study had two related objectives: first, to determine whether the computationally prioritized compounds differed in toxicity and behavioural effects in zebrafish, and, second, to examine the selected compound in mouse behavioural and molecular assays. The comparison was intended to identify a candidate for further mechanistic work rather than to establish SERT inhibition or therapeutic efficacy.

## 2. Materials and Methods

### 2.1. Ethics, Reporting Standards and Data Availability

Animal work in this study was conducted within a formal inter-institutional research program between South Ural State University (SUSU) and the Institute of Cytology and Genetics, Siberian Branch of the Russian Academy of Sciences (ICG SB RAS). Research Agreement No. 025/09-01 was signed by the two institutions on 15 July 2025, before the experimental work. Under this collaborative framework, the mouse experimental arm was performed at the Centre for Genetic Resources of Laboratory Animals, ICG SB RAS, Novosibirsk, and was approved by the Ethics Committee of ICG SB RAS (protocol No. 241, 29 July 2025). The zebrafish experiments were performed at SUSU as the aquatic-vertebrate screening component of the same collaborative scientific program. No separate SUSU zebrafish ethics protocol was issued. The term “affiliated laboratory” used in an earlier revision was descriptive rather than a formal institutional designation and has therefore been removed. No a priori power calculation was documented; group sizes followed established laboratory protocols and were constrained by the available cohorts, and the study is interpreted as exploratory. Endpoint-specific sample sizes and exclusions are reported in the Results, figure legends and tables. Reporting was prepared with reference to ARRIVE 2.0, with non-randomized procedures and unavailable methodological information reported explicitly, rather than reconstructed retrospectively [17]. Embryos were allocated sequentially and were not randomized; mouse groups were balanced for age and body weight but were not formally randomized; the adult zebrafish allocation method was not documented. The investigator conducting mouse behavioural testing was unaware of group assignment. Formal blinding of dosing personnel and data analysts, and formal group-coding procedures for other experimental stages, were not documented. Automated tracking or instrument-based measurement is not presented as equivalent to formal blinding.

### 2.2. Bioinformatic Analysis

In silico SERT-oriented prioritization was based on the previously published multitarget modelling program [18,19]. The SERT-specific source dataset comprised 3436 known compounds and drugs with reported SERT-inhibitory activity. The published workflow identified 21 relevant target proteins and 25 binding sites, retained 68 valid three-dimensional target models, and used ensemble docking to generate neural-network input descriptors. In the SERT-affinity modelling stage, 7500 regression neural networks were trained; the selected model used 24 binding sites across 20 relevant targets and showed an overall correlation coefficient RTotal = 0.885 (*p* < 5 × 10^−7^) [18]. AutoDock Vina 1.1.1 was used as the docking engine [20].

(1) $$\mathrm{Cons}=\overset{7}{\underset{i=1}{\Sigma }}{\mathrm{Ind}}_{i}$$

The consensus-classification component used seven neural-network predictions. Consensus aggregation followed the previously published seven-network ensemble methodology, in which alternative train/test/validation partitions were generated at a 5:1:1 ratio, and a positive consensus required at least four concordant positive predictions among seven networks [19]. For the compounds examined here, the retained outputs were the consensus-level Cons (the number of positive high-activity predictions; 4–7 for a positive classification) and the membership function Fm for the high-activity class (0–1; typically >0.2 for compounds assigned to the active class).

(2) $$F_{m}=\frac{1}{7}\overset{7}{\underset{i=1}{\Sigma }}F_{i}\cdot {\mathrm{Ind}}_{i}$$

These published validation characteristics document the provenance and internal performance of the computational framework; they do not constitute experimental validation of SERT binding for the present imidazo[1,2-a]pyridine-oxadiazolopyrazine series. No scaffold-specific external binding or uptake validation and no meaningful scaffold-level positive-rate estimate are available for this three-compound, preselected series. The classifier outputs are therefore treated only as SERT-oriented prioritization metrics, not as direct binding or uptake evidence.

### 2.3. Compound-Code Harmonization and Compound Selection

Before assembly, compound identifiers were harmonized across the computational, chemical and biological datasets. The code lock used throughout is as follows: AD20 = UtIA-0108 = compound **3b** = Gr2-09; AD19 = UtIA-0167 = compound **3a** = Gr2-07; and AD12 = UtIA-0111 = compound **3c** = Gr2-16. During code harmonization, AD20 was fixed as UtIA-0108/**3b**/Gr2-09; an obsolete UtIA-0138 label present in earlier working materials was not used for biological interpretation. Duplicate structures in the prediction files were treated as code synonyms rather than independent compounds. For biological follow-up, AD19/UtIA-0167 and AD20/UtIA-0108 were evaluated in zebrafish assays, and AD20/UtIA-0108 was subsequently evaluated in mouse behavioural and molecular follow-up; AD12/UtIA-0111 was retained as a computational/chemical reserve candidate because no zebrafish or mouse data are available for it in the available dataset.

### 2.4. Chemical Synthesis and Compound Characterization

The target imidazo[1,2-a]pyridine derivatives **3a–c** were prepared by TiO_2_-photocatalysed aerobic oxidative C–H/C–H coupling of imidazo[1,2-a]pyridines with [1,2,5]oxadiazolo[3,4-b]pyrazines, following the methodological framework reported by Utepova and co-workers [21,22]. Reactions were performed in a quartz vessel under irradiation with a Xe lamp (5000 K, 35 W) in refluxing acetic acid, affording compounds **3a–c** in 24–37% yield. Full NMR spectra are provided in the Supplementary Materials (Figures S1–S6).

**Scheme 1:**
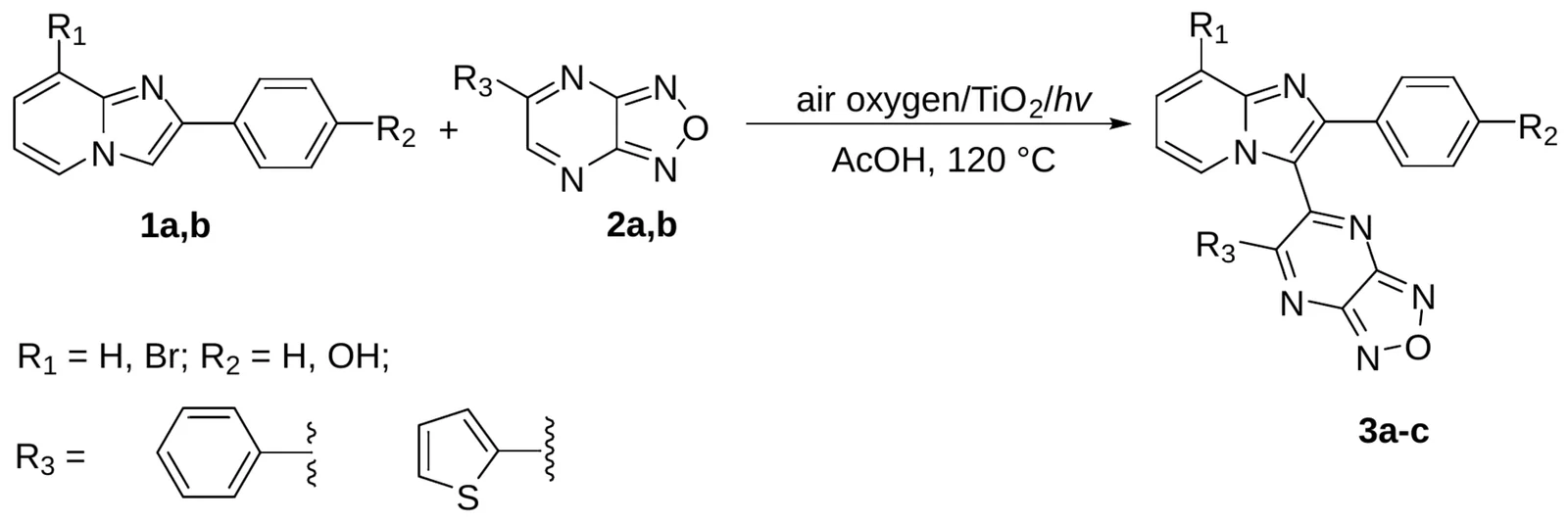
TiO_2_-photocatalysed C–H/C–H coupling of imidazo[1,2-a]pyridines with [1,2,5]oxadiazolo[3,4-b]pyrazines, affording compounds **3a–c**.

The target compounds **3a–c** were prepared by TiO_2_-photocatalysed C–H/C–H coupling, as shown in Scheme 1.

General experimental methods. ^1^H-NMR (400 MHz) and ^13^C-NMR (101 MHz) spectra were recorded on an Avance II instrument (Bruker, Ettlingen, Germany); ^1^H-NMR (600 MHz) and ^13^C-NMR (150 MHz) spectra were recorded on an Avance III instrument (Bruker, Germany) in DMSO-d6 or CDCl_3_, using SiMe_4_ as internal reference. Chemical shifts (δ) are reported in ppm and coupling constants (J) in Hz. EI mass spectra were acquired by direct inlet on a Shimadzu GCMS-QP2010 Ultra in full-scan mode. The ion-source temperature was 200 °C; the interface temperature was 150 °C for **3a** and **3b** and 250 °C for **3c**; scan ranges were *m*/*z* 35–550 for **3a** and **3b** and *m*/*z* 35–750 for **3c**, at a scan speed of 5000. High-resolution spectra were acquired on a maXis impact Q-TOF mass spectrometer (Bruker Daltonics) using negative-ion electrospray in full-scan mode (*m*/*z* 50–3300; capillary 4500 V; end-plate offset −100 V; charging voltage 2000 V; nebulizer 0.2 bar; dry gas 4.0 L/min; dry heater 200 °C). Tandem MS/MS was not performed; therefore, collision energy is not applicable. Reactions were monitored by TLC on 0.25 mm silica-gel plates (60 F254). Silica gel 60 (0.04–0.063 mm, Macherey-Nagel, Düren, Germany) was used for column chromatography. Melting points were determined on an SMP10 apparatus (Stuart, Stone, Staffordshire, UK) and are uncorrected. Imidazo[1,2-a]pyridines **1a,b** and 5-(hetero)aryl[1,2,5]oxadiazolo[3,4-b]pyrazines **2a,b** were prepared according to published procedures [23,24].

General procedure for the synthesis of compounds **3a**, **3b** and **3c**. A quartz tube containing a solution of imidazo[1,2-a]pyridine **1a,b** (0.5 mmol) and 5-(hetero)aryl[1,2,5]oxadiazolo[3,4-b]pyrazine **2a,b** (0.5 mmol) in acetic acid (20 mL) with TiO_2_ (10 mass %, anatase) was treated in an ultrasonic bath for 3 min to obtain a suspension. The mixture was exposed to a Xe lamp (5000 K, 35 W) under air (oxygen bubbled through the mixture) and kept boiling for 6 h. The mixture was concentrated under reduced pressure and the residue purified by column chromatography on silica gel.

5-Phenyl-6-(2-phenyl-8-bromoimidazo[1,2-a]pyridin-3-yl)[1,2,5]oxadiazolo[3,4-b]pyrazine (**3a**; AD19, UtIA-0167, Gr2-07) was purified by column chromatography (hexane/ethyl acetate 9:1), yielding a dark-red solid (70 mg, 30%), mp 238–240 °C, R_f_ = 0.1. ^1^H NMR (600 MHz, CDCl_3_) δ 9.19 (d, J = 6.8, 1H, 5′-H), 7.77 (d, J = 7.4, 1H, 7′-H), 7.34–7.18 (m, 2H, Ph and Ph’), 7.12 (t, J = 7.5, 2H, Ph), 7.05–6.97 (m, 3H, 6′-H, Ph’), 6.93 (d, J = 7.5, 2H, Ph’), 6.87 (d, J = 7.6, 2H, Ph). ^13^C NMR (150 MHz, CDCl3) δ 163.93, 153.68, 152.20, 150.89, 150.77, 145.45, 136.48, 133.33, 130.70, 130.42, 128.97, 128.75, 128.71, 128.10, 128.03, 125.08, 119.46, 114.33, 112.09. MS (EI): *m*/*z* 470 (base peak). HRMS (ESI^−^): *m*/*z* calcd for C_23_H_13_BrN_6_O (79Br isotopologue) 468.0334; found 468.0342. The corresponding 81Br isotopic signal was observed at *m*/*z* 470.0325 (calcd 470.0314).

5-(Thien-2-yl)-6-(2-phenyl-8-bromoimidazo[1,2-a]pyridin-3-yl)[1,2,5]oxadiazolo[3,4-b]pyrazine (**3b**; AD20, UtIA-0108, Gr2-09). The compound was purified by column chromatography (benzene) to yield a dark-red solid (57 mg, 24%), mp 228–230 °C, R_f_ = 0.1. ^1^H NMR (600 MHz, CDCl_3_) δ 8.93 (d, J = 6.7, 1H, 5′-H), 7.78 (d, J = 7.3, 1H, 7′-H), 7.37 (d, J = 4.7, 1H, thiophene), 7.29–7.27 (m, 1H, Ph’), 7.22–7.20 (m, 4H, Ph’), 6.98 (t, J = 7.3, 1H, 6′-H), 6.95 (m, J = 4.7, 1H, thiophene), 6.78 (t, J = 4.3, 1H, thiophene). ^13^C NMR (150 MHz, CDCl3) δ 156.80, 152.81, 151.91, 150.84, 150.61, 145.35, 140.19, 133.87, 133.13, 131.75, 130.59, 129.13, 128.78, 128.68, 127.90, 124.83, 119.04, 114.34, 112.14. MS (EI): *m*/*z* 476 (base peak). HRMS (ESI^−^): *m*/*z* calcd for C_21_H_11_BrN_6_OS 475.9878; found 475.9893.

5-Phenyl-6-(2-(4-hydroxyphenyl)imidazo[1,2-a]pyridin-3-yl)[1,2,5]oxadiazolo[3,4-b]pyrazine (**3c**; AD12, UtIA-0111, Gr2-16) was purified by column chromatography (hexane/ethyl acetate 7:3), yielding a dark-red solid (75 mg, 37%), mp 285–287 °C, R_f_ = 0.2. ^1^H NMR (400 MHz, DMSO-d6) δ 9.60 (s, 1H, OH), 9.03 (d, J = 6.9, 1H, 8′-H), 7.74 (d, J = 9.0, 1H, 5′-H), 7.59–7.47 (m, 1H, 7′-H), 7.23 (t, J = 7.4, 1H, 6′-H), 7.16 (t, J = 6.7, 1H, Ph’), 7.03 (t, J = 7.8, 2H, Ph), 6.95 (d, J = 8.2, 4H, Ph’), 6.55 (d, J = 8.5, 2H, Ph). ^13^C NMR (101 MHz, DMSO-d6) δ 165.18, 158.59, 155.16, 152.06, 151.18, 150.34, 146.80, 137.10, 130.50, 130.28, 128.62, 128.52, 127.65, 126.78, 124.91, 117.34, 117.22, 115.66, 113.89. MS (EI): *m*/*z* 406 (base peak). HRMS (ESI^−^): *m*/*z* calcd for C_23_H_14_N_6_O_2_ [M−H]^−^ 405.1100; found 405.1105.

Copies of the ^1^H and ^13^C NMR spectra, MS, and HRMS data are provided in the Supplementary Materials (Figures S1–S12).

### 2.5. Zebrafish Embryo Acute-Toxicity Assay

Embryo toxicity was evaluated over 96 h in an adapted design based on the plate layout and four apical lethality observations of OECD TG 236 [25]. Newly fertilized eggs were washed and immersed in the respective test solutions as soon as possible after fertilization and no later than 90 min post-fertilization. Before the 16-cell stage, viable fertilized embryos showing synchronous cleavage, an intact chorion and no obvious cleavage abnormalities were selected under a stereomicroscope and transferred to covered, flat-bottom 24-well plates containing 2 mL of solution per well. Embryos were allocated sequentially by well position rather than by randomization. Each concentration comprised 20 embryos, one embryo per well, with four internal dilution-water controls on the same plate. Separate plates contained 20 solvent-control embryos, 20 positive-control embryos and 24 dilution-water negative-control embryos. AD19 and AD20 were tested in separate main experiments with separate controls; each main experiment was preceded by pilot range finding, which was not treated as an independent replicate. The positive control was 3,4-dichloroaniline at 4 mg/L. Reconstituted dilution water contained CaCl_2_·2H_2_O (294.0 mg/L), MgSO_4_·7H_2_O (123.3 mg/L), NaHCO_3_ (64.7 mg/L) and KCl (5.7 mg/L) in distilled water. During exposure, pH was 7.4–7.6, dissolved oxygen remained above 80% saturation, total hardness was 250–260 mg/L as CaCO_3_, and temperature was 26 °C under a 12 h light/12 h dark cycle. AD20 was prepared as a 0.2 mg/mL stock in 1% aqueous DMSO and diluted without further DMSO addition. Nominal concentrations of 0.05, 0.03, 0.02, 0.01, 0.005 and 0.001 mg/mL contained 0.25%, 0.15%, 0.10%, 0.05%, 0.025% and 0.005% DMSO, respectively. Solutions were visually clear, without visible precipitate or turbidity, and no additional solvent or formulation component was used. Embryos were examined at 24, 48, 72 and 96 h for coagulation, lack of somite formation, non-detachment of the tail and lack of heartbeat; any positive finding was classified as death. Hatching and gross developmental abnormalities were recorded, the same embryo was followed over time, and coagulated embryos were removed after observation.

### 2.6. Larval Zebrafish Light–Dark Locomotion Assay

Abrupt changes in illumination elicit reproducible locomotor responses in zebrafish larvae, but these responses are not specific measures of anxiety. Drug-related changes may reflect altered sensorimotor processing, arousal, sedation, toxicity, or other neuroactive effects. The light–dark assay was therefore used here as a locomotor phenotyping screen rather than as a selective anxiolytic-efficacy test [26].

The effect of the candidate compounds on the locomotor activity of *Danio rerio* larvae was assessed following the protocol described in [27] using the ZebraBox^®^ tracking system (ViewPoint Life Sciences, Lyon, France). The ZebraBox records movement by automated video capture with an infrared camera (25 frames per second), enabling continuous recording of the whole plate under both light and dark conditions. ZebraLab 3 software (ViewPoint Life Sciences, Lyon, France) was used to program the light-cycle protocol and to analyze larval behavioural activity.

Eggs were treated with 5% sodium hypochlorite for 5 min and rinsed in 500 mL of embryo medium.

Fertilized embryos (*n* = 30 per group) were distributed into Petri dishes filled with control water—defined as aerated fish-system water passed through a multistage filtration train (coarse mechanical, 50 µm, biological, carbon, and UV stages; pH 7.3 ± 0.05, conductivity 750 ± 50 µs/cm, dissolved O_2_ 90 ± 5%).

Based on the embryo-toxicity results, the larval test concentrations were 5 µg/mL AD20/UtIA-0108, 1 µg/mL AD19/UtIA-0167, and 2.5 µg/mL imipramine hydrochloride. These were toxicity-constrained screening concentrations rather than concentrations established as broadly safe or therapeutically relevant.

**Figure 1:**
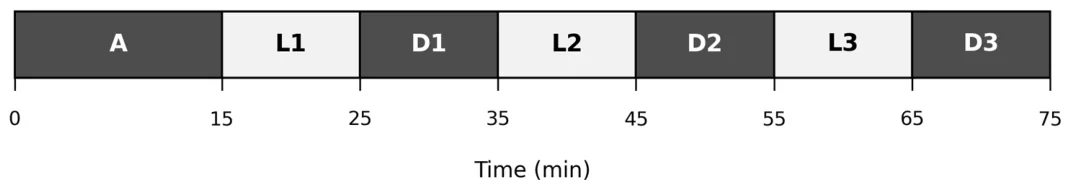
Larval light–dark locomotion protocol. A, 15 min dark adaptation; L1, L2, L3 (light) and D1, D2, D3 (dark), alternating 10 min periods (total 75 min).

At 6 days post-fertilization, larvae were placed individually into 96-well plates containing the test solutions (one larva per well; *n* = 24 for control, imipramine and AD20, and *n* = 20 for AD19 in the analyzed dataset). Exposure began 20 min before locomotor recording and did not begin at the embryo stage. Each well contained 250 µL of solution and was filled to approximately two-thirds of its height, leaving an air–water interface for swim-bladder inflation [28]. The protocol comprised 15 min of dark acclimation followed by three alternating 10 min light and 10 min dark periods (Figure 1).

Testing was performed between 13:00 and 15:00 [29]. The ZebraBox used the programmed full-light setting during light phases and infrared recording during dark phases; illuminance at plate level was not recorded. Low-speed (<3 mm/s), medium-speed (3–6 mm/s), and high-speed (>6 mm/s) movement bands were analyzed. Acclimation data were displayed for context but were not used for load-bearing inference.

Because the test concentrations were close to the embryo-toxicity boundaries, the assay was interpreted strictly as a neuroactivity and locomotor-state screen. Period-specific and pooled light/dark comparisons were exploratory; the results were not used as standalone evidence of anxiolytic or antidepressant activity. The mortality percentages observed at these nominal concentrations in the embryo assay arose from 96 h exposure beginning shortly after fertilization and should not be interpreted as expected mortality rates during the short 6-dpf larval exposure. The retained records do not contain dedicated survival, malformation, or heart-rate measurements for the behavioural cohort, and no lower-dose behavioural series was performed; consequently, a subtoxic contribution cannot be excluded. Imipramine at 2.5 µg/mL is therefore retained only as a qualitative reference comparator rather than a quantitative efficacy benchmark [30].

### 2.7. Adult Zebrafish Chronic Unpredictable Stress and Novel-Tank Testing

Adult wild-type short-fin zebrafish (3–5 months old) were obtained from a commercial distributor and maintained in a recirculating system with mechanical, chemical and biological filtration and UV sterilization (25 °C, pH 7.2–7.5, ammonia < 0.9 mg/L, nitrate < 200 mg/L; 14/10 h light/dark, lights on at 08:00; fed dry food and Artemia twice daily). The source draft reported 240 adult fish at a 1:1 male:female ratio. A 14-day chronic unpredictable stress (CUS) protocol was used as the stress-context model [31], combining predator exposure, spatial restriction, alarm pheromone, food deprivation, shallow-water stress, light stress, social isolation, net chasing, mild electric stimulation, hypothermia, and other changes in stress modality. The day-by-day schedule is given in Table 1.

Adult fish were assigned to eight groups: control, CUS, control+imipramine, control+AD20/UtIA-0108, control+AD19/UtIA-0167, CUS+imipramine, CUS+AD20/UtIA-0108 and CUS+AD19/UtIA-0167. The acute dataset contained 10 fish per group (80 total). The retained chronic dataset contained 158 fish, with group sizes of 20 (CUS), 20 (CUS+imipramine), 16 (CUS+AD19), 18 (CUS+AD20), 24 (control), 24 (control+imipramine), 19 (control+AD19) and 17 (control+AD20). The source workbook did not document the reasons for the lower chronic group counts, so no post hoc exclusion mechanism is inferred. Adult zebrafish exposure concentrations were the same as those used in the larval screen: AD20/UtIA-0108 5 µg/mL (0.005 mg/mL), AD19/UtIA-0167 1 µg/mL (0.001 mg/mL), and imipramine hydrochloride 2.5 µg/mL (0.0025 mg/mL). Acute exposure consisted of one 20 min treatment before a single novel-tank session; chronic exposure consisted of daily 3 h treatment at the same assigned concentration for 14 days followed by testing 24 h after the final exposure. Sex was balanced across groups and pooled in the analysis. The retained records do not document a formal randomization procedure for adult fish allocation.

The novel-tank apparatus was a narrow glass tank reported as 20 cm long × 15 cm high × 5 cm wide. Testing began no earlier than 2 h after lights-on. Each fish was introduced individually into the filled tank and recorded for 5 min; water was replaced between trials.

The recorded video was processed automatically with EthoVision XT17 (Noldus IT, Wageningen, The Netherlands), yielding the frequency, cumulative duration (s) and latency (s) of entry into each zone, the distance travelled (m), the mean swimming velocity (m/s) and the maximum acceleration (m/s^2^). The tank was divided into three zones: upper (1/4 of the height), middle (2/4), and lower (1/4).

Whole-body cortisol measurements were available for the chronic adult zebrafish cohort; however, the retained records did not preserve sufficient tissue-extraction and assay-platform details for full procedural replication. The cortisol dataset is therefore reported only as exploratory Supplementary Information (Table S2) and is not used for primary inference.

### 2.8. Mouse Animals, Drug Administration and Behavioural Testing

Mouse experiments were performed using adult male ASC/Icg (Antidepressant Sensitive Cataleptics) mice (P60), a selectively bred strain developed at the Institute of Cytology and Genetics, SB RAS, from CBA × (CBA × AKR) backcrosses involving the catalepsy-prone CBA/Lac and catalepsy-resistant AKR/J parental strains [32]. Animals were housed under SPF conditions at the Centre for Genetic Resources of Laboratory Animals, ICG SB RAS, under a 12 h light/12 h dark cycle with food and water ad libitum. Groups were balanced for age and body weight, but they were not formally randomized. The investigator conducting behavioural testing was unaware of group assignment; formal blinding of dosing personnel and data analysts was not documented. AD20/UtIA-0108 was dissolved in 100% DMSO and diluted with sterile deionized water to 4% DMSO (*v*/*v*). Imipramine hydrochloride was prepared in the same vehicle, and controls received 4% DMSO. Formulation pH and a quantitative solubility limit were not determined; preparations were inspected visually and microscopically for turbidity or precipitate before administration.

For acute testing, imipramine hydrochloride was administered intraperitoneally at 25 mg/kg and AD20/UtIA-0108 at 5 or 20 mg/kg. Because AD20 had not previously been characterized in mice and had no established pharmacologically active dose or pharmacokinetic exposure range, 5 and 20 mg/kg were used as exploratory acute doses. The lower dose of 5 mg/kg was carried forward into the repeated-administration arm. The retained records do not document a more specific pharmacokinetic or efficacy-based rationale for that choice; importantly, the choice was not based on an acute efficacy signal, as neither acute dose produced an AD20-versus-vehicle behavioural effect. No formal pharmacokinetic dose conversion or dedicated dose-ranging study was performed. The imipramine regimens were used as laboratory reference conditions consistent with prior experimental use of imipramine in genetically catalepsy-prone mice [33]. The open-field test was performed 1 h after acute administration, the forced-swim test was performed 1 h later, and mice were euthanized 1 h after completion of the forced-swim procedure. Catalepsy was assessed in a separate acute series 2 h after administration. For chronic testing, imipramine at 15 mg/kg, AD20/UtIA-0108 at 5 mg/kg, or vehicle was administered intraperitoneally once daily for 21 days. After treatment day 16, mice were individually housed for 2 days while dosing continued. The open-field, forced-swim immobility, and light–dark box procedures were performed on days 19, 20, and 21, respectively; the daily injection was given after each session. The final injection followed the day-21 test, and mice were euthanized 24 h later. Catalepsy was assessed on day 14.

Open-field test. Testing was conducted for 5 min in a circular arena 40 cm in diameter with a 25 cm high white wall. The semitransparent floor was illuminated from below by two 12 W halogen lamps positioned 40 cm beneath it. Each mouse was placed near the wall; distance travelled and time in the centre were quantified with EthoStudio. The arena was cleaned with 70% ethanol between trials.

Light–dark box. The apparatus measured 45 × 27 × 27 cm and contained illuminated and dark compartments, which were separated by an opaque partition with a 7 × 7 cm opening. The light compartment was illuminated by a 300 W lamp. Each mouse was placed in the light compartment facing the opening, and latency to enter the dark compartment and time in the light compartment were recorded for 5 min. The apparatus was cleaned with 70% ethanol between animals.

Forced-swim immobility endpoint. Each mouse was placed in a clear 30 × 30 × 30 cm vessel containing water at 25 °C. After 2 min of adaptation, mobility and immobility were automatically for 4 min with EthoStudio, based on frame-to-frame changes in the animal’s silhouette. This endpoint is reported as forced-swim immobility and is not interpreted as a direct measure of antidepressant efficacy.

Catalepsy. Under natural lighting between 12:00 and 16:00, the mouse was held by the scruff for 5 s and placed on parallel bars with the forepaws 5 cm above the hind paws. Latency was measured until either forepaw moved or a gross body/head movement occurred, up to 120 s. Immobility exceeding 20 s was scored as positive; trials were repeated at 2 min intervals until three positive responses were obtained. Catalepsy results were reported descriptively because raw categorical counts were unavailable.

### 2.9. Brain-Tissue Processing and Quantitative PCR

Frontal cortex, hippocampus and midbrain samples from the same animals were used for qPCR and HPLC by splitting each homogenate. Frozen tissue was homogenized for 10–20 s in 300 µL of 50 mM Tris-HCl (pH 7.6) at 4 °C. A 250 µL aliquot was mixed with MagZol Reagent for RNA extraction, and the remaining 50 µL was mixed with 200 µL of 0.6 M HClO_4_ for HPLC. Samples were dissected on ice, frozen in liquid nitrogen and stored at −80 °C. The frontal cortex was defined as a 2–3 mm section anterior to the corpus callosum after removal of the olfactory bulbs; the hippocampus was separated from subcortical structures and rolled away from the cortex; the midbrain/raphe-containing sample was bounded anteriorly by the superior colliculi and posteriorly by the rhomboid fossa, after removal of the superior colliculi.

Total RNA was isolated from the 250 µL aliquot with MagZol Reagent (Guangzhou Magen Biotechnology, Guangzhou, China), treated with RNase-free DNase I, assessed by NanoDrop spectrophotometry (Wilmington, Delaware, United States), adjusted to 125 ng/µL and stored at −80 °C. Reverse transcription used random hexamers and M-MuLV reverse transcriptase at 41 °C for 60 min.

Real-time PCR was performed using a Roche LightCycler 96 (Basel, Switzerland) with SYBR Green/ROX chemistry in 20 µL reactions. Samples were analyzed in technical duplicate, no-template controls were included, and melting curves were generated after each run (Table 2). Cycling comprised 94 °C for 3 min followed by 40 cycles of 94 °C for 10 s, gene-specific annealing for 30 s and 72 °C for 30 s. A genomic-DNA dilution series (0.125–128 ng/µL) generated calibration curves. Expression was reported as target-gene cDNA copies per 100 copies of Polr2a cDNA [34,35,36]. The full primer panel is provided in Supplementary Table S1.

### 2.10. HPLC Analysis of Monoamines and the 5-HIAA/5-HT Ratio

A 50 µL aliquot of homogenate was mixed with 200 µL of 0.6 M HClO_4_ containing isoproterenol (200 ng/mL) as an internal standard, centrifuged at 12,000× *g* for 15 min at 4 °C, diluted twofold with ultrapure water and filtered through a 0.22 µm cellulose-acetate Spin-X filter. Twenty microlitres were injected into a Shimadzu LC-20AD/DGU-20A5R/SIL-20A/CBM-20A HPLC system (Shimadzu Corporation, Kyoto, Japan) coupled to a DECADE II electrochemical detector (Antec Leyden B.V., Zoeterwoude, the Netherlands), operated at 750 mV with a VT-03 3-mm glassy-carbon flow cell (Antec Leyden B.V., Zoeterwoude, the Netherlands). Separation was isocratic at 0.6 mL/min and 40 °C on a Luna C18 (Phenomenex, Torrance, CA, USA) column (75 × 4.6 mm, 5 µm) with a C8 guard cartridge. The mobile phase comprised 90% 50 mM phosphate buffer (pH 3.9) containing sodium 1-octanesulfonate as ion-pair reagent and 10% methanol. Serotonin and 5-HIAA were expressed as ng/mg protein; their ratio was dimensionless. One acute midbrain ratio value (0.018545) was source-flagged and excluded by the laboratory’s ROUT procedure.

### 2.11. Protein Determination

The residual protein pellet was stored at −20 °C, diluted tenfold in 0.1 M NaOH to 50 µL and incubated at 37 °C for 15 min. Standards (0–1000 µg/mL) and samples were analyzed in duplicate. Bradford reagent (Bio-Rad, Feldkirchen, Germany), diluted fivefold, was added at 150 µL per well; after 15 min, absorbance was read at 595 nm on a Multiscan plate reader (Thermo Fisher Scientific, Waltham, MA, USA).

### 2.12. Statistical Analysis

Statistical analyses used GraphPad Prism 9.1.0 and the analysis procedures specified for each experimental layer. Fish behavioural figures are presented as mean ± SEM; whereas, mouse HPLC and qPCR summaries are reported as mean ± SD. No a priori power calculation was documented, and post hoc observed power calculations were not used. Embryo mortality is reported as observed n/N within one main concentration series per compound; the original probit LC50 values are retained only for exploratory within-study ranking. Larval light–dark measurements are treated as repeated observations from the same larvae and are displayed primarily as descriptive period-specific and pooled-light/dark summaries; pairwise tests are exploratory and not used for efficacy claims. Adult novel-tank zone-duration contrasts were Holm-corrected across the three specified zone-duration endpoints, with locomotor covariates examined by analysis of covariance as described in the Results. Mouse distributional assumptions were assessed with the D’Agostino–Pearson test. Depending on distribution, one-way ANOVA or Kruskal–Wallis tests were followed by vehicle-controlled and AD20-versus-imipramine comparisons; qPCR multiplicity was controlled using Benjamini–Hochberg FDR within condition × region gene families. ROUT (Q = 5%) was applied per endpoint in the source mouse workbooks; it was not documented as pre-specified. Across the mouse workbooks, 19 endpoint values in the acute dataset and 37 in the chronic dataset (56 total) were source-flagged, and all final *n* values are reported per endpoint. Samples unavailable because of method-specific or preparation failure were omitted without imputation. No mouse behavioural endpoint was documented as a prespecified primary outcome; the chronic light–dark latency finding is therefore treated as exploratory. For the reviewer-requested effect-size reporting, the retained individual-level latency data were summarized with group medians and Hedges’ g, and a 95% confidence interval for the between-group mean difference was calculated using an unequal-variance (Welch) approach.

## 3. Results

Results are presented in the order in which the compounds were evaluated: identity and computational prioritization, chemical characterization, zebrafish embryo toxicity, larval locomotion, adult zebrafish behaviour, and mouse behavioural and molecular findings. Computational predictions were used to select compounds for testing; final prioritization was based on the combined experimental results.

### 3.1. Code Correction and Compound Identity

**Figure 2:**
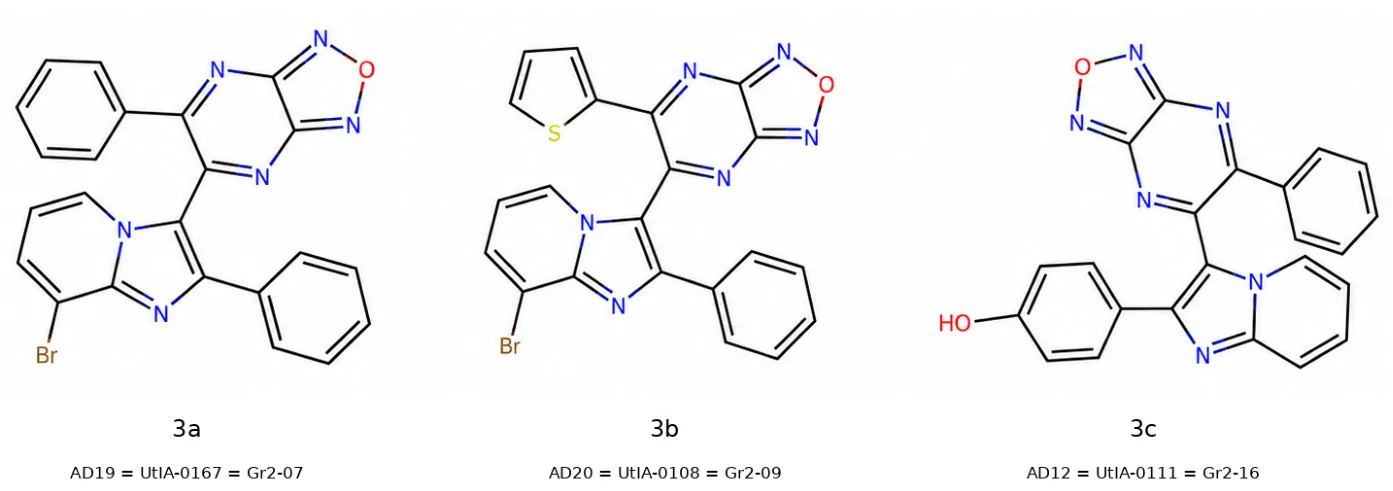
Chemical structures and harmonized compound identities of the prioritized imidazo[1,2-a]pyridine–[1,2,5]oxadiazolo[3,4-b]pyrazine candidates. The figure shows the article-wide numbering and code relationships used throughout the manuscript: **3a** = AD19 = UtIA-0167 = Gr2-07; **3b** = AD20 = UtIA-0108 = Gr2-09; and **3c** = AD12 = UtIA-0111 = Gr2-16.

Before biological interpretation, all compound codes were reconciled against the computational prediction files, the chemistry article codes, and the chemist-supplied cross-check tables. The corrected mapping used in all analyses is AD20 = UtIA-0108 = **3b** = Gr2-09; AD19 = UtIA-0167 = **3a** = Gr2-07; and AD12 = UtIA-0111 = **3c** = Gr2-16 (Figure 2).

Table 3 summarizes the locked compound identities, computational prioritization metrics, and current biological status of the three compounds included in the study. All three compounds were classified in the high SERT-oriented prediction class, but their prioritization metrics were not concordant: AD19/UtIA-0167 showed the strongest consensus level, AD20/UtIA-0108 an intermediate-high consensus level, and AD12/UtIA-0111 the highest membership-function value despite having no in vivo data in the current study. This non-concordance supports the staged triage logic, in which computational ranking was treated as an entry filter rather than as a final candidate-prioritization criterion. The classifier output should be read as a SERT-oriented prioritization signal, not as evidence that these compounds are experimentally proven SERT inhibitors.

ConsLev denotes the number of positive high-activity votes among seven neural-network classifiers; FCm(h) denotes the membership-function value for the high-activity class. The consensus level and FCm(h) are the only computational prioritization outputs reported in the source data for the tested compounds. The SERT-oriented prediction is a classifier output and must not be interpreted as direct experimental evidence of SERT binding or serotonin-transporter inhibition. Molecular formulae were confirmed by HRMS (Supplementary Materials).

### 3.2. Computational Prioritization Identifies Three Candidates with High Predicted Activity but Does Not Establish SERT Inhibition

The consensus classifier assigned all three compounds to the high predicted-activity class. AD19/UtIA-0167 showed the highest consensus level, with seven positive votes and FCm(h) = 0.435. AD20/UtIA-0108 showed six positive votes and FCm(h) = 0.457. AD12/UtIA-0111 showed five positive votes and FCm(h) = 0.499.

**Figure 3:**
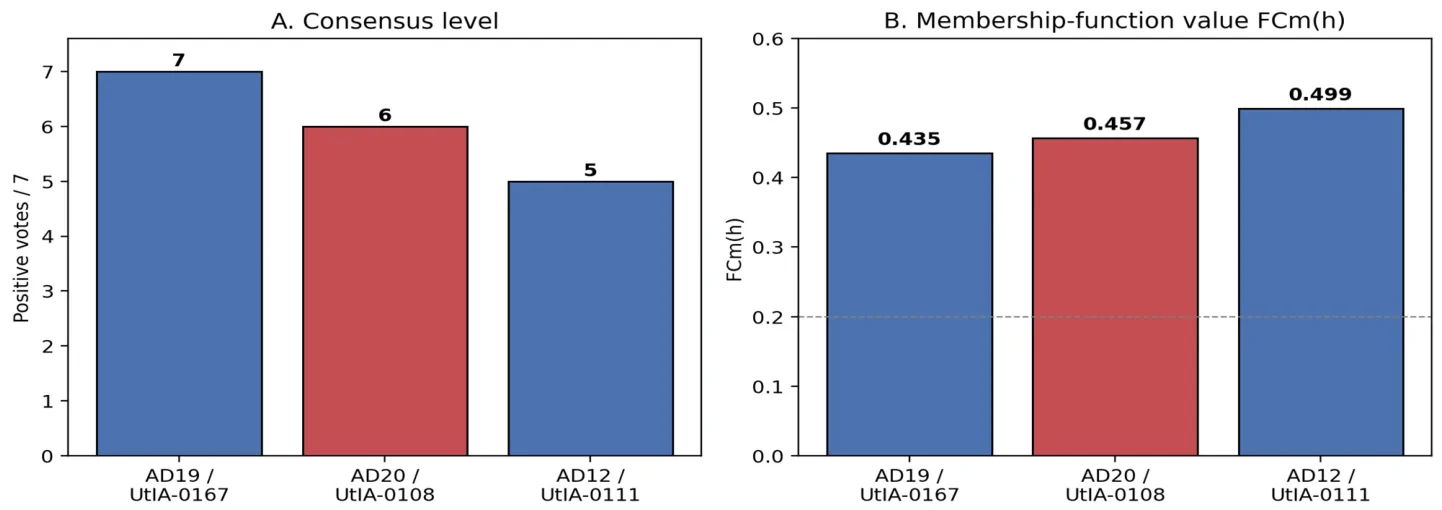
Computational prioritization of the candidate series. The consensus classifier assigned all three compounds to the high predicted-activity class; the consensus level (number of positive votes among the seven neural-network models) and the membership-function value FCm(h) are shown for each compound. These are SERT-oriented prioritization metrics and do not establish direct target engagement. AD20/UtIA-0108 was advanced as the prioritized biological candidate on the basis of the downstream zebrafish and mouse triage rather than the highest computational consensus.

The ranking therefore depends on the metric used. By consensus level, the order is AD19/UtIA-0167 > AD20/UtIA-0108 > AD12/UtIA-0111. By FCm(h), however, the order is AD12/UtIA-0111 > AD20/UtIA-0108 > AD19/UtIA-0167 (Figure 3). This divergence argues against treating the computational output as a single deterministic candidate-prioritization result and supports the staged triage design. Because no direct SERT uptake or binding assay is available, these results are reported as SERT-oriented computational prioritization rather than experimental confirmation of SERT inhibition.

### 3.3. Chemical Identity and Structural Interpretation

Chemically, the three candidates are rigid polyheteroaryl compounds rather than classical SERT inhibitor-like molecules with an obvious protonatable aliphatic amine. AD19/UtIA-0167 is an 8-bromo phenyl-substituted imidazo[1,2-a]pyridine–oxadiazolopyrazine compound [37,38]; AD20/UtIA-0108 is the corresponding 8-bromo thien-2-yl analogue; and AD12/UtIA-0111 contains a hydroxyphenyl substituent.

This structural profile is consistent with a non-classical CNS-active heteroaromatic chemotype, but it does not support direct transfer of the canonical tricyclic-antidepressant/selective-serotonin-reuptake-inhibitor (TCA/SSRI) pharmacophore logic to these compounds. In particular, the absence of an obvious protonatable aliphatic amine makes a classical protonated-amine transporter-binding explanation insufficient without direct binding or uptake experiments. The immediate prioritization decision was therefore not based on the strongest computational consensus alone: AD19/UtIA-0167 remains the highest-consensus predicted candidate but is biologically deprioritized; AD20/UtIA-0108 remains the prioritized candidate because it survived the staged zebrafish triage and entered exploratory mouse follow-up; and AD12/UtIA-0111 remains a computational/chemical reserve candidate because the available dataset contains no in vivo data for this compound. Limited practical solubility also argued against including it in the zebrafish exposure workflow at this stage.

### 3.4. Zebrafish Embryo Toxicity Defines the Exposure Boundary for Behavioural Testing

In the main AD20/UtIA-0108 embryo series, mortality was 100% at 0.05 and 0.03 mg/mL, 70% at 0.02 mg/mL, 55% at 0.01 mg/mL, 35% at 0.005 mg/mL and 10% at 0.001 mg/mL. Hatching was observed in 14/20 embryos at 0.001 mg/mL. The 0.25% DMSO solvent control showed 3/20 deaths, all recorded by 24 h, and 17/20 embryos hatched. Additional solvent-control observations at 0.15% and 0.10% DMSO showed two deaths and zero deaths, respectively. The dilution-water negative control showed 1/24 mortality, with all 23 surviving embryos hatching by 96 h; whereas, the 4 mg/L 3,4-dichloroaniline positive control showed 19/20 mortality. An exploratory probit fit yielded the previously reported LC50 of 0.0110 mg/mL. Because DMSO concentration varied across the series and mortality in the highest DMSO control was 15%, this value is used only as a descriptive toxicity-ranking estimate and not as a guideline-valid regulatory LC50.

**Figure 4:**
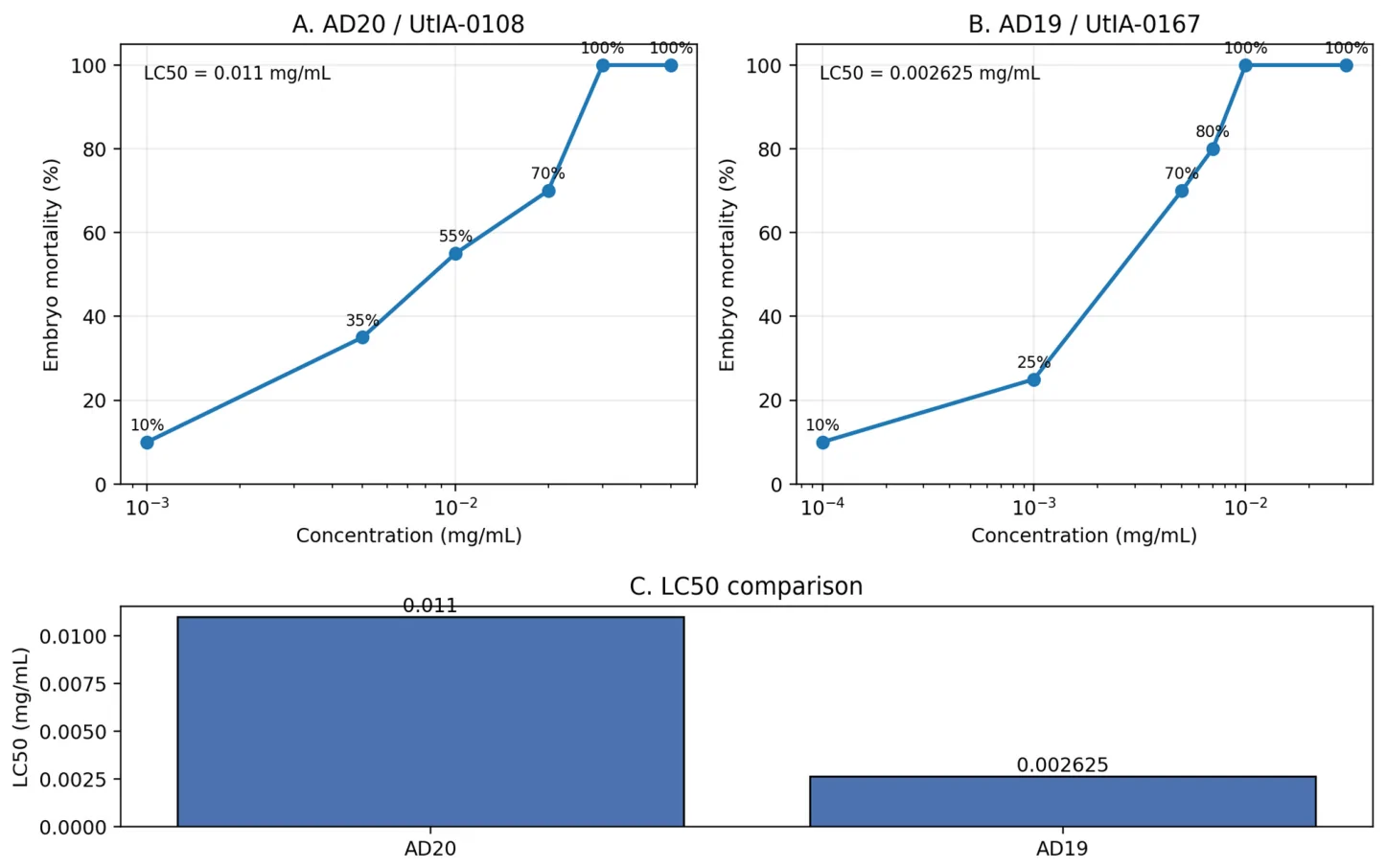
Exploratory zebrafish embryo-toxicity profiles used to define exposure boundaries for subsequent behavioural testing. Mortality was recorded in one main 96 h series per compound after pilot range finding, with 20 embryos per nominal concentration. The original probit LC50 estimates are shown as descriptive ranking values. The experiment used an adapted OECD TG 236 design, but it is not interpreted as guideline-valid because the vehicle concentration varied across the AD20 series and the 0.25% DMSO control showed 15% mortality.

AD19/UtIA-0167 showed a narrower exposure range. Mortality was 100% at 0.03 and 0.01 mg/mL, 80% at 0.007 mg/mL, 70% at 0.005 mg/mL, 25% at 0.001 mg/mL and 10% at 0.0001 mg/mL; solvent-control mortality was 10%. The original exploratory probit LC50 estimate was 0.002625 mg/mL. The 4 mg/L 3,4-dichloroaniline positive control produced 18/20 deaths. These findings were used to constrain later exposure selection and to rank AD19 below AD20 on embryo toxicity; they are not presented as a regulatory toxicity study (Figure 4).

### 3.5. Larval Light–Dark Locomotion Indicates Neuroactive Locomotor Modulation Under Toxicity-Constrained Exposure

Automated ZebraBox/ZebraLab tracking was performed under a light–dark protocol consisting of a 15 min dark acclimation period followed by three 10 min light phases and three 10 min dark phases. Larval locomotion was analyzed in three speed bands: low-speed (<3 mm/s), medium-speed (3–6 mm/s) and high-speed (>6 mm/s). The acclimation period is shown for completeness but was not included in the statistical comparisons.

During the light periods, the most consistent treatment-related changes were observed in the low-speed range (<3 mm/s), and the interpretation was therefore centred on this band. Nominal medium-speed light-period changes (AD20/UtIA-0108 frequency *p* = 0.013 and duration *p* = 0.033; imipramine distance *p* = 0.031) were considered exploratory. AD20/UtIA-0108 increased the frequency of low-speed episodes (1.7-fold; *p* = 0.0036) and the total low-speed distance (1.8-fold; *p* = 0.0031) relative to control. AD19/UtIA-0167 increased low-speed episode duration (1.5-fold; *p* = 0.020) and low-speed distance (2.0-fold; *p* = 0.0002). Imipramine increased low-speed episode duration (1.6-fold; *p* = 0.0007) and low-speed distance (1.6-fold; *p* = 0.0052). In direct comparison with imipramine, AD20/UtIA-0108 produced more frequent but shorter low-speed episodes (frequency *p* = 0.0016; duration *p* = 0.0024), indicating that the two treatments produced different larval profiles.

In the pooled dark periods, all treated groups shifted away from reactive locomotor activation. Low-speed episode duration increased relative to control by approximately 2.1-fold for AD20/UtIA-0108 (*p* = 0.0006), 2.6-fold for AD19/UtIA-0167 (*p* < 0.0001) and 2.9-fold for imipramine (*p* < 0.0001), and low-speed distance increased 1.9-fold (*p* = 0.0015), 2.0-fold (*p* = 0.0003) and 1.7-fold (*p* = 0.0049), respectively; low-speed episode frequency fell only in the imipramine group (approximately 2-fold; *p* = 0.040). In the medium-speed band (3–6 mm/s), episode frequency, duration and distance were reduced in all treated groups, most strongly for imipramine (frequency reduced approximately 7.5-fold, *p* = 0.0001) and AD20/UtIA-0108 (approximately 3-fold, *p* = 0.0055); for AD19/UtIA-0167 the medium-speed reduction was significant for frequency (*p* = 0.041) and distance (*p* = 0.001) but only nominal for duration (*p* = 0.056). In the high-speed band (>6 mm/s), AD20/UtIA-0108 (frequency *p* = 0.011; duration *p* = 0.021; distance *p* = 0.011) and imipramine (all *p* < 0.002) reduced locomotion, whereas AD19/UtIA-0167 did not reach significance (all *p* > 0.07). Across the dark period, AD20/UtIA-0108 again differed from imipramine, with more frequent but shorter low-speed episodes (*p* = 0.0006 and *p* = 0.0002).

**Figure 5:**
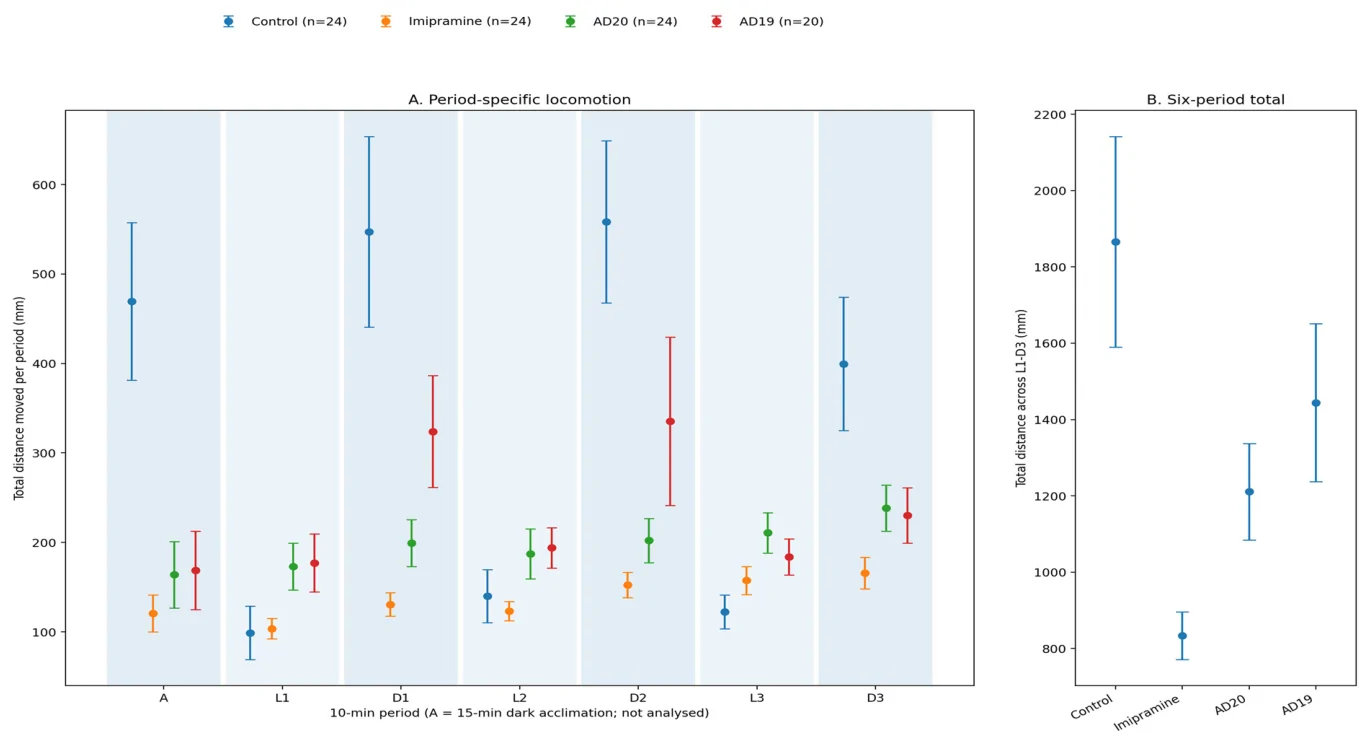
Larval zebrafish light–dark locomotion. (**A**) Period-specific total distance moved (mean ± SEM) during the 15 min dark acclimation and three alternating light and dark periods; observations are shown without connecting lines because they are discrete repeated periods. (**B**) Total distance across the six analyzed periods (L1–D3). AD20/UtIA-0108 and imipramine showed marked suppression of dark-period activation; whereas, AD19/UtIA-0167 was intermediate. *n* = 24 for control, imipramine, and AD20; *n* = 20 for AD19. The display is descriptive and is not interpreted as evidence of anxiolysis.

Taken together, the treatments produced distinguishable locomotor-state profiles rather than a selective anxiety readout (Figure 5). AD20/UtIA-0108 and imipramine markedly reduced dark-period activation, while AD19/UtIA-0167 produced an intermediate pattern. At the concentration used, imipramine largely suppressed the normal light–dark response, indicating that sedation or generalized locomotor suppression contributed to the comparator profile. The uncorrected pairwise comparisons are therefore retained only for descriptive phenotypic triage and do not establish anxiolysis, antidepressant-like efficacy, or SERT engagement.

### 3.6. Adult Zebrafish CUS/Novel-Tank Triage Separates AD19/UtIA-0167 from AD20/UtIA-0108

Adult novel-tank (NTT) behaviour was analyzed using the three-zone partition specified by the experimenters: upper quarter Top (1_4), lower quarter Bottom (1_4), and middle half Mid (2_4). Fish were tested in the NTT after either acute or chronic compound exposure on a chronic unpredictable stress (CUS) background. Throughout, “acute” and “chronic” refer to the duration of compound exposure; the novel-tank test itself was always a single session. The acute-exposure analysis included 80 fish and the chronic-exposure analysis 158 fish across the eight stress-by-treatment groups.

**Figure 6:**
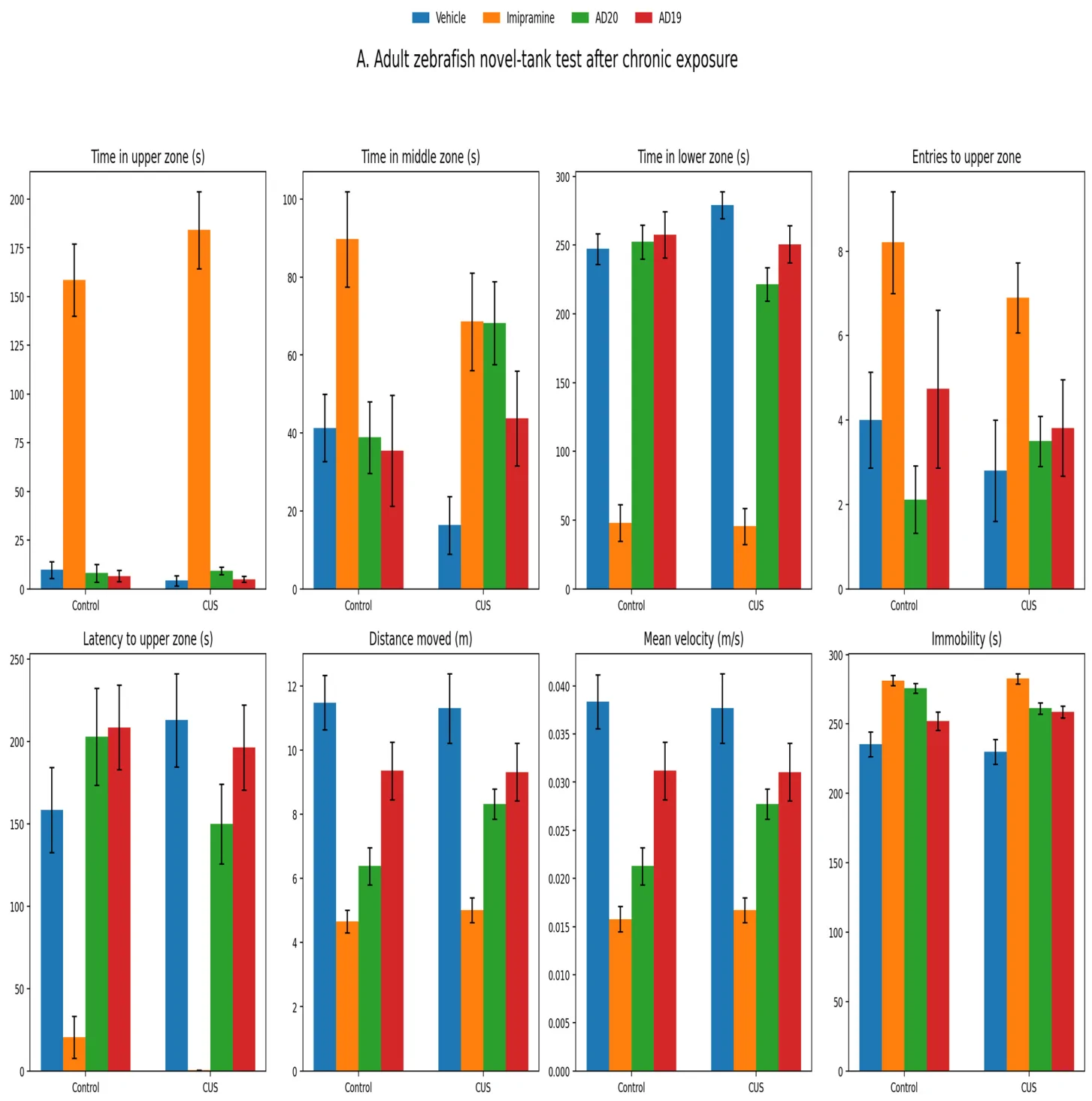

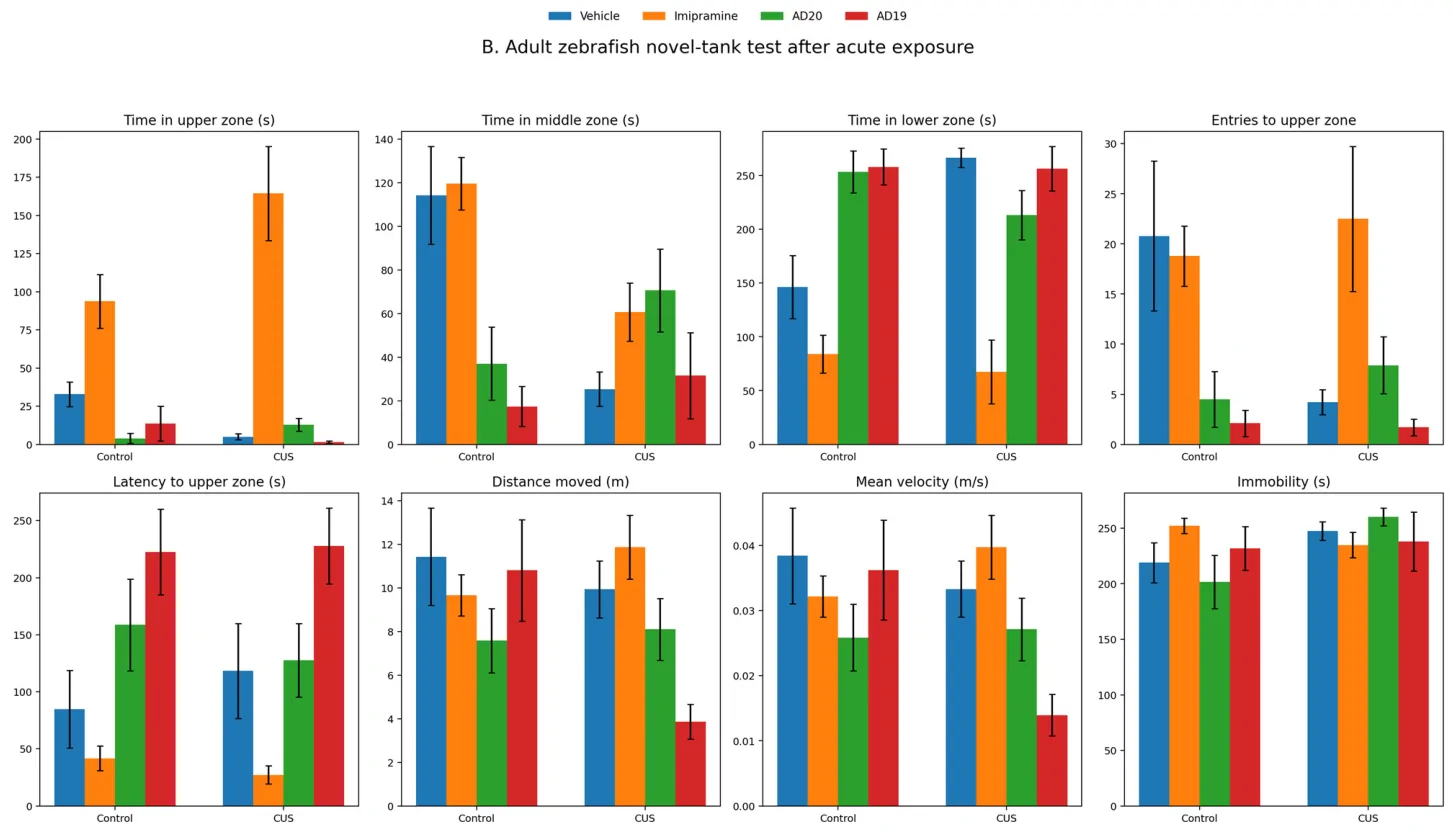
Adult zebrafish novel-tank test after (**A**) chronic and (**B**) acute compound exposure. Bars show mean ± SEM for the eight stress-by-treatment groups. The acute dataset contained 10 fish per group. Chronic group sizes were 16–24, as specified in the Methods. The figure is descriptive; Holm-corrected zone-duration contrasts and the locomotor-covariate analysis used for interpretation are reported in the Results section.

After acute compound exposure, AD20/UtIA-0108 did not produce an anxiolytic-like or rescue-like profile: relative to the stress vehicle, stress+AD20 did not differ on any of the three zone-duration endpoints (middle zone *p* = 0.089; lower zone *p* = 0.076; upper zone *p* = 0.36). AD19/UtIA-0167 was likewise unfavourable after acute exposure. The novel-tank test after acute exposure therefore provided no positive efficacy signal in adult zebrafish for AD20/UtIA-0108 (Figure 6).

After chronic CUS and chronic compound exposure, imipramine served as the reference anxiolytic and validated the assay by increasing upper- and middle-zone exploration and reducing lower-zone occupancy in stressed fish. The dominant CUS effect in this cohort was a loss of middle-zone occupancy: chronic unpredictable stress reduced middle-zone duration from 41.3 s in control to 16.3 s in stress vehicle; whereas, it raised lower-zone duration only modestly (control 247.3 s, stress vehicle 279.1 s) and the upper-zone change was small.

Against this background, the chronic AD20/UtIA-0108 effect was centred on recovery of middle-zone occupancy: relative to the stress vehicle, stress+AD20 restored middle-zone duration to 68.3 s (Holm *p* = 0.001, across the three prespecified zone-duration endpoints). Lower-zone occupancy was a secondary, concordant readout, reduced to 221.7 s (Holm *p* = 0.001); because CUS itself raised the lower zone only modestly, this is described as reduced lower-zone occupancy rather than reversal of a strong stress-induced bottom dominance. The upper-zone increase (4.2 to 9.2 s, Holm *p* = 0.007) was nominal and is not load-bearing, and frequency and latency measures provided no additional corrected AD20-positive signal; the adult-Danio claim is therefore duration-based and centred on middle-zone recovery (Figure 6).

Chronic stress+AD20 was accompanied by reduced general locomotor activity and increased immobility relative to stress vehicle; in an analysis of covariance adjusting each zone’s cumulative duration for total distance moved and immobility, the middle-zone increase (adjusted increase 54.2 s, *p* = 0.0004) and the lower-zone reduction (adjusted reduction 59.5 s, *p* = 0.0012) persisted, with parallel regression slopes across groups and a concordant rank-based analysis of covariance. The zone redistribution was therefore not explained by the reduced locomotor activity or the increased immobility, although these covariates are themselves treatment-affected and the analysis should not be interpreted as causal deconfounding; the direction of the effect is consistent with this interpretation, because a simple sinking or sedation artefact would increase rather than decrease lower-zone occupancy. The chronic AD20/UtIA-0108 effect is accordingly described as stress-context attenuation, dominated by middle-zone recovery, and not as full normalization or an imipramine-like profile. AD19/UtIA-0167 did not show this profile: under the three-zone analysis it did not differ from control in the unstressed condition and produced only nominal stressed-fish shifts that did not survive correction; together with its greater embryo toxicity, this makes AD19/UtIA-0167 the less favourable candidate, and it should not be described as uniformly anxiogenic.

Exploratory whole-body cortisol data from the chronic adult zebrafish cohort are provided in Supplementary Table S2. Because complete tissue-extraction and assay-platform details were not available for full procedural replication, these data were not used for the principal interpretation of the adult zebrafish experiment.

### 3.7. Exploratory Mouse Behavioural Follow-Up of AD20/UtIA-0108

AD20/UtIA-0108 was advanced to the mouse stage as the compound was retained after the preceding computational, chemical, and zebrafish triage. The mouse experiments are interpreted as exploratory mammalian follow-up of the prioritized candidate, not as independent efficacy validation or as a parallel mammalian screen; AD19/UtIA-0167 and AD12/UtIA-0111 were not tested in the available mouse dataset.

In the acute experiment, AD20/UtIA-0108 did not produce a robust antidepressant-like or locomotor profile relative to vehicle. In the open-field test, the omnibus effect for travelled distance was significant, but the AD20 5 mg/kg and 20 mg/kg groups did not differ from DMSO in the planned vehicle-controlled comparisons (Dunnett-type *p* = 0.9807 and *p* = 0.9709). AD20 also did not reduce forced-swim immobility relative to DMSO (5 mg/kg, *p* = 0.9837; 20 mg/kg, *p* = 0.8527). These acute data do not support an imipramine-like antidepressant profile for AD20/UtIA-0108 in mice.

The clearest behavioural signal emerged after chronic AD20/UtIA-0108 treatment in the light–dark box. AD20 significantly increased the latency to enter the dark compartment relative to DMSO (DMSO 11.87 ± 4.47 s, *n* = 11; AD20 27.06 ± 3.84 s, *n* = 13; Kruskal–Wallis *p* = 0.0062; AD20 vs. DMSO *p* = 0.0101). AD20 also differed from imipramine on this endpoint (AD20 vs. imipramine *p* = 0.0112), indicating that the profile should not be presented as simply imipramine-like. This latency endpoint was exploratory and was not documented as a pre-specified primary outcome. In the retained individual-level data, the median latency was 26.5 s with AD20 and 5.6 s with DMSO. The mean difference was 15.19 s (95% CI, 2.92 to 27.46 s), with Hedges’ g = 1.02.

This chronic effect was endpoint-specific. The percentage of time in the light compartment did not differ among groups (one-way ANOVA *p* = 0.3866; AD20 vs. DMSO *p* = 0.2894), and chronic AD20 did not affect open-field travelled distance (*p* = 0.6014), open-field centre time (*p* = 0.6456) or forced-swim immobility (*p* = 0.9210). The mouse behavioural data therefore identify an endpoint-specific, preliminary anxiolytic-like signal based on entry latency rather than a broad antidepressant- or anxiolytic-like phenotype; this finding was not replicated in an independent anxiety paradigm and requires prospective replication. Catalepsy was evaluated as a safety-oriented motor/extrapyramidal endpoint on treatment day 14; because the available catalepsy data are summarized as percentages without raw counts, no formal categorical inference is reported and the catalepsy data remain descriptive.

### 3.8. Mouse Monoamine-Metabolism and qPCR Readouts

Acute midbrain 5-HIAA/5-HT did not differ significantly between AD20 and vehicle groups; therefore, acute HPLC did not support an AD20-specific serotonergic-turnover effect.

A secondary, region-specific HPLC signal was observed after chronic treatment in the hippocampus. The hippocampal 5-HIAA/5-HT ratio was lower in AD20 than in DMSO (DMSO 0.830 ± 0.038, *n* = 11; AD20 0.733 ± 0.014, *n* = 11; Kruskal–Wallis *p* = 0.0011; AD20 vs. DMSO *p* = 0.0409; AD20 vs. imipramine *p* = 0.000218). Chronic cortical and midbrain ratios did not show significant differences between AD20 and DMSO. Absolute 5-HT and 5-HIAA concentrations are reported separately in Table 4, while the dimensionless 5-HIAA/5-HT ratio is retained as the turnover index. The analyte-level values are descriptive and do not provide additional evidence of SERT engagement.

Values are presented as mean ± SD. n denotes the number of included endpoint measurements after source-flagged exclusions. 5-HT—serotonin; 5-HIAA—5-hydroxyindoleacetic acid; DMSO—dimethyl sulfoxide.

**Figure 7:**
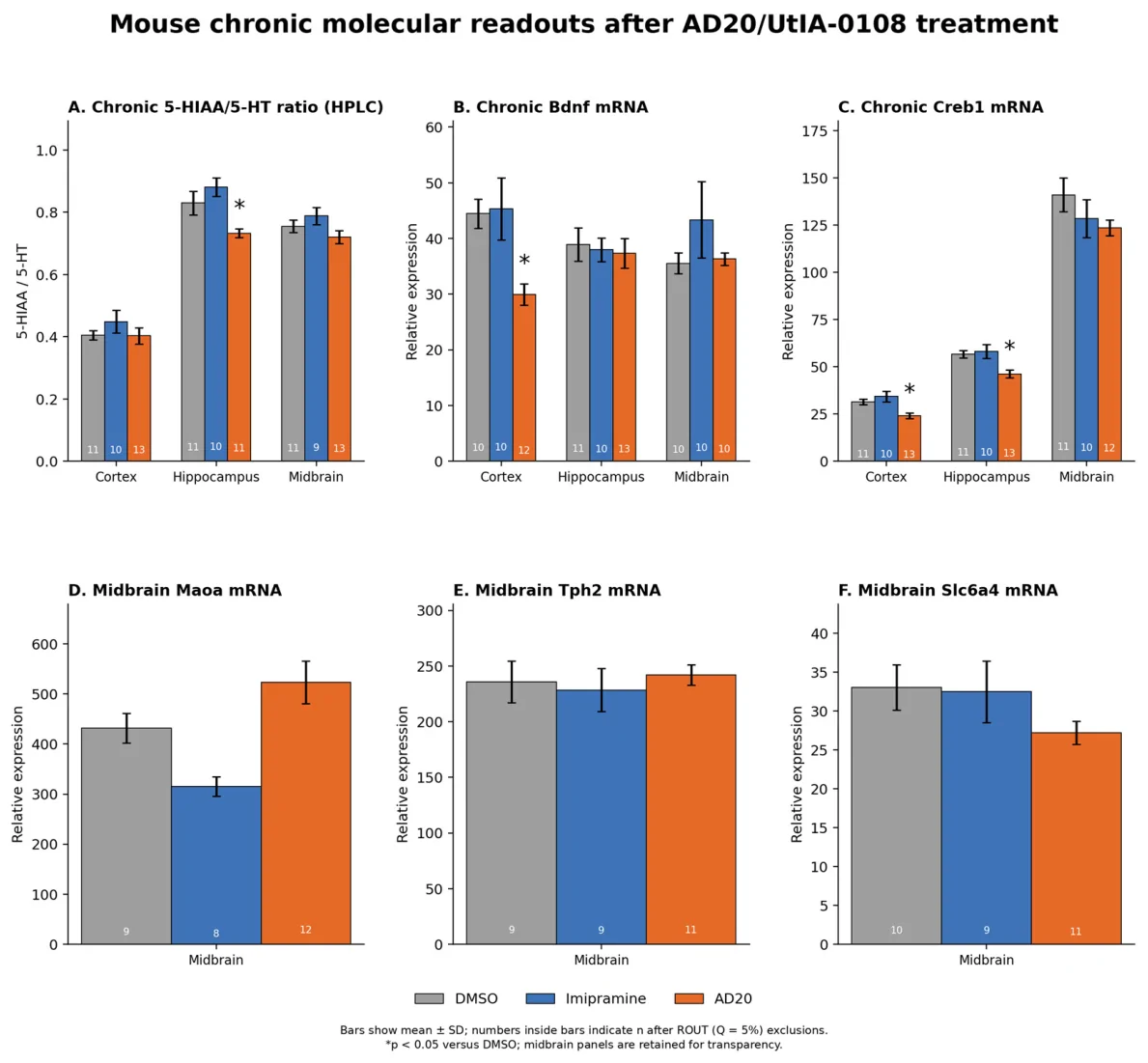
Mouse chronic molecular findings for AD20/UtIA-0108. Chronic treatment effects on serotonin turnover and qPCR readouts were analyzed in the cortex, hippocampus, and midbrain of ASC/Icg mice. Bars show mean ± SD; numbers inside bars indicate n after ROUT 5% exclusions. AD20/UtIA-0108 significantly decreased the hippocampal 5-HIAA/5-HT ratio and reduced Bdnf and Creb1 expression in selected forebrain regions. Midbrain data are shown for transparency and did not support a significant AD20-versus-vehicle effect on 5-HIAA/5-HT, Creb1, Maoa, Tph2 or Slc6a4. * *p* < 0.05 versus DMSO vehicle according to the source/recomputed statistics.

The qPCR data showed the most consistent AD20-associated changes in *Bdnf*/*Creb1*-related readouts after chronic treatment. In the cortex, AD20 was associated with lower *Bdnf* (DMSO 44.47 ± 2.60, *n* = 10; AD20 29.95 ± 1.89, *n* = 12; ANOVA *p* = 0.0053; FDR *p* = 0.0105; AD20 vs. DMSO *p* = 0.0120) and lower *Creb1* (DMSO 31.44 ± 1.39, *n* = 11; AD20 24.06 ± 1.50, *n* = 13; ANOVA *p* = 0.0014; FDR *p* = 0.0055; AD20 vs. DMSO *p* = 0.0146). A parallel *Creb1* signal was observed in the hippocampus (DMSO 56.74 ± 1.94, *n* = 11; AD20 46.23 ± 2.12, *n* = 13; ANOVA *p* = 0.0039; FDR *p* = 0.0155; AD20 vs. DMSO *p* = 0.0112); whereas, hippocampal *Bdnf* did not differ among groups (*p* = 0.9106) (Figure 7).

The midbrain *Maoa* results require cautious interpretation. Although the midbrain *Maoa* omnibus/FDR result was significant (ANOVA *p* = 0.0016; FDR *p* = 0.0110), the AD20-versus-DMSO contrast was not significant (*p* = 0.1287) and the pattern was driven mainly by the AD20-versus-imipramine contrast (*p* = 0.000463). The manuscript therefore does not state that AD20 changed midbrain *Maoa* expression relative to vehicle. Midbrain *Slc6a4* and *Tph2* showed no significant AD20-versus-DMSO effects, further arguing against interpreting the mouse molecular data as evidence of direct SERT-target engagement. Together, the molecular readouts are best presented as region-specific downstream correlates of a restricted anxiolytic-like behavioural signal, not as proof of SERT inhibition.

## 4. Discussion

AD20/UtIA-0108 was selected from a SERT-oriented computational screen, but the experimental findings do not demonstrate SERT inhibition. The clearest comparison is with AD19/UtIA-0167. AD19 received the strongest computational score, yet it was more toxic to zebrafish embryos and did not produce a consistent adult zebrafish behavioural effect. AD20 had a wider usable concentration range and produced measurable effects in adult zebrafish and, subsequently, in mice. This divergence shows why the computational ranking was used for initial selection rather than as the principal evidence for prioritizing candidates.

The zebrafish experiments provided two distinct types of information. The embryo assay showed that AD19 was more toxic than AD20, although interpretation of the AD20 concentration–mortality series is limited by variable DMSO concentrations and 15% mortality in the highest solvent control. In larvae, AD20 and imipramine both reduced dark-period activity, but the near-complete suppression of the light–dark response by imipramine indicates that generalized locomotor suppression contributed to this pattern. The larval experiment therefore demonstrates neuroactivity rather than a specific anxiolytic effect. In chronically stressed adult fish, AD20 increased middle-zone occupancy and reduced lower-zone occupancy. These changes persisted after adjustment for distance moved and immobility, but they did not amount to full normalization and were not reproduced after acute exposure. Importantly, the 35% mortality for AD20 at 5 µg/mL and 25% mortality for AD19 at 1 µg/mL arose from the separate 96 h embryo exposure; whereas, the behavioural cohort consisted of 6-dpf larvae exposed only 20 min before a 75 min recording. Those embryo mortality percentages therefore cannot be interpreted as expected mortality during the short larval behavioural exposure. Nevertheless, dedicated larval survival, malformation, and heart-rate measurements were not collected for the behavioural cohort, and no lower-dose behavioural series was available; a subtoxic contribution to the locomotor phenotype therefore cannot be excluded.

The zebrafish and mouse findings should not be interpreted as evidence for a shared SERT mechanism. Zebrafish possess two serotonin-transporter paralogues, *slc6a4a/serta* and *slc6a4b/sertb*; whereas, mice have a single SERT orthologue [12,13,39]. Moreover, ligand recognition differs between zebrafish and mammalian SERT, including for imipramine-related compounds [14]. Waterborne exposure also provides no direct estimate of systemic or brain exposure [40]. Thus, the zebrafish experiments were useful for toxicity and phenotypic selection, but they cannot predict the affinity of AD20 for mouse or human SERT. Studies using paralogue-specific zebrafish models and direct transporter assays are needed to resolve this point.

Structure–activity relationship hypothesis. AD20 lacks the protonatable aliphatic amine present in many high-affinity SSRIs and tricyclic antidepressants [41,42,43,44]. Its rigid polyheteroaryl scaffold could support aromatic or hydrophobic interactions, but the present computational analysis does not define a binding mode. The behavioural and molecular findings are also compatible with actions at other monoaminergic or non-monoaminergic targets. Direct binding and uptake assays, followed by broader target profiling, are therefore required before the compound can be assigned a transporter-based mechanism. Within the closely related AD19–AD20 pair, replacement of the phenyl substituent in AD19 by a thien-2-yl group in AD20 was associated with a wider embryo-toxicity window and a more favourable chronic adult zebrafish phenotype. This two-compound comparison is a structure–phenotype association rather than a resolved mechanistic SAR, because the present data cannot distinguish changes in target affinity from differences in physicochemical properties, uptake, distribution, or metabolism. A broader analogue series is required to test this relationship.

In mice, AD20 produced one positive behavioural result after chronic treatment: latency to enter the dark compartment was increased. Time in the light compartment, open-field distance and centre time, and forced-swim immobility were unchanged. The effect should therefore be described as endpoint-specific and preliminary; altered sensory processing or response initiation cannot be excluded. The reduction in hippocampal 5-HIAA/5-HT and the changes in *Bdnf* and *Creb1* expression were region-specific and did not coincide with changes in *Slc6a4* or *Tph2*. These molecular findings may accompany the behavioural effect, but they do not identify its mechanism. The direction of the cortical Bdnf and Creb1 changes is not a canonical antidepressant-like molecular signature. These transcriptional changes are therefore treated as region-specific downstream correlates of uncertain functional significance rather than as mechanistic support for antidepressant efficacy.

The study has several limitations. Direct SERT binding and uptake, pharmacokinetics and brain exposure were not measured. The embryo assay included one main concentration series per compound, and the AD20 series had variable DMSO concentrations; the reported LC50 is therefore descriptive. The larval behavioural concentrations were toxicity-constrained; dedicated sublethal endpoints were not collected in that cohort, and no lower-dose behavioural series was available. Exploratory cortisol data were not used for mechanistic or efficacy inference because complete assay documentation was unavailable. Embryo allocation was sequential rather than randomized; mouse groups were balanced for age and body weight but not formally randomized; and the adult-fish allocation method and blinding of several experimental stages were not documented. The mouse behavioural finding was exploratory, confined to one light–dark endpoint and not confirmed in a second anxiety paradigm. Further work should begin with direct transporter and broader target assays, measurement of internal exposure, and prospective independent replication of the mouse behavioural finding.

## 5. Conclusions

AD20/UtIA-0108 was selected for further study because it showed lower embryo toxicity than AD19/UtIA-0167, measurable effects in chronically stressed adult zebrafish and an exploratory endpoint-specific effect in the mouse light–dark box. The data do not demonstrate direct SERT inhibition or a general antidepressant- or anxiolytic-like profile. Region-specific HPLC and qPCR changes provide additional biological information but do not establish a mechanism.

AD20 should therefore be considered an early neuroactive candidate rather than a validated SERT inhibitor. The absence of a canonical protonatable amine, differences between zebrafish and mammalian SERT, and the limited mouse behavioural evidence all argue for a broader pharmacological assessment. Direct binding and uptake studies, pharmacokinetic measurements, and independent behavioural replication are required before target-specific or therapeutic conclusions can be drawn.

## Figures and Tables

**Table 1:** Fourteen-day chronic unpredictable stress (CUS) schedule for adult zebrafish.

CUS Day	Stressor Sequence and Duration
**1**	Predator stress (two adult 7 cm cichlids, *Cichlasoma nigrofasciatum*, for 8 h) → 12 h light deprivation (complete darkness) in home tanks.
**2**	Spatial restriction and novelty stress (2 L plastic containers, stocking density 7–11 fish/L) for 8 h with alarm pheromone (30 mL added) → transfer to home tank.
**3**	24 h food deprivation, alarm pheromone (30 mL) and addition of 2 L of water from a predatory-fish tank.
**4**	8 h exposure in a 4 L light–dark tank → shallow-water stress (50% of normal level) + light stress (2.6–2.7 klux) for 2 h → 16 h combination of predator chemical cues (1 L predator water) and food deprivation → return to home tank.
**5**	Repeated aversive mild electric current for 1 min, three times at 5 min intervals, in a novel environment → return to home tank.
**6**	Social isolation in 100 mL opaque cups for 8 h → 30% water change in the home tank (3 L) + 16 h complete darkness.
**7**	Shaking for 6 h (22 °C, 60 rpm) in home tanks with 40 mL alarm-pheromone extract + light stress (2.6–2.7 klux) → transfer to home tank.
**8**	Repeated aversive mild electric current for 1 min, three times at 5 min intervals, in a novel environment.
**9**	Removal from water for 1 min, three times at 10 min intervals → predator stress for 5 h → alarm pheromone (10 mL, 7 times over 15 min) → transfer to home tank.
**10**	Spatial-restriction stress + sensory stimulation (green, red and yellow containers, 2 h each) → 3 L predator-tank water added to the home tank.
**11**	8 h exposure under crowding (2 L tank), shallow water (5 cm) and constant illumination → 2 L predator-tank water added to the home tank.
**12**	Net chasing for 15 min → spatial restriction in a 4 L tank + light stress (17.4–17.8 klux) for 20 min → hypothermia (0.5 L ice added) → shallow-water stress (30%) for 12 h in the home tank.
**13**	Shallow-water stress (30% of normal level, 3 L) for 18 h + alarm pheromone (100 mL, 5 times over 15 min).
**14**	Predator stress for 8 h → 12 h light deprivation (complete darkness) in home tanks.

CUS—chronic unpredictable stress. Arrows indicate sequential stressors applied within the same day.

**Table 2:** qPCR primer sequences and amplicon characteristics for mouse gene expression analysis.

Gene	Forward Primer (5′ → 3′)	Reverse Primer (5′ → 3′)	Annealing Temperature (°C)	Amplicon Length (bp)
*Bdnf*	tacctggatgccgcaaacat	tgcttcagttggcctttgga	60	191
*Creb1*	gctggctaacaatggtacggat	tggttgctgggcactagaat	64	140
*Maoa*	aatgaggatgttaaatgggtagatgttggt	cttgacatattcaactagacgctc	63	138
*Slc6a4*	cgctctactacctcatctcctcc	gtcctgggcgaagtagttgg	63	101
*Tph2*	cattcctcgcacaattccagtcg	agtctacatccatcccaactgctg	61	239
Polr2a (reference gene)	tgtgacaactccatacaatgc	ctctcttagtgaatttgcgtact	60	194

Primer sequences are shown in the 5′ → 3′ direction. Polr2a was used as the reference gene. The extended primer panel is provided in Supplementary Table S1.

**Table 3:** Compound identity, SERT-oriented prediction metrics, embryo-toxicity boundary, and cross-species prioritization status.

Attribute	AD19/UtIA-0167	AD20/UtIA-0108	AD12/UtIA-0111
UtIA code	UtIA-0167	UtIA-0108	UtIA-0111
Article/group code	**3a**/Gr2-07	**3b**/Gr2-09	**3c**/Gr2-16
Substituent (scaffold)	8-Br, phenyl	8-Br, thien-2-yl	4-hydroxyphenyl (no 8-Br)
Molecular formula	C_23_H_13_BrN_6_O	C_21_H_11_BrN_6_OS	C_23_H_14_N_6_O_2_
Predicted class	High	High	High
Consensus level (0–7)	7	6	5
FCm(h)	0.435	0.457	0.499
Embryo LC50 (mg/mL)	0.002625	0.0110	not determined (no in vivo data)
Cross-species prioritization	Deprioritized despite highest consensus (greater embryo toxicity; unfavourable adult NTT profile)	Prioritized early neuroactive candidate (zebrafish + exploratory mouse follow-up)	Computational/chemical reserve; no in vivo data

Abbreviations: LC50—median lethal concentration; NTT—novel-tank test; SERT—serotonin transporter. Computational scores are prioritization metrics and do not constitute experimental evidence of SERT inhibition.

**Table 4:** Absolute 5-HT and 5-HIAA concentrations in mouse brain regions after chronic treatment.

Brain Region	Treatment Group	5-HT (ng/mg Protein)	n	5-HIAA (ng/mg Protein)	n
Cortex	DMSO	3.81 ± 2.32	11	1.54 ± 0.96	11
Cortex	Imipramine	3.41 ± 1.62	10	1.44 ± 0.44	10
Cortex	AD20	2.87 ± 0.32	12	1.14 ± 0.17	13
Hippocampus	DMSO	2.72 ± 0.37	10	2.32 ± 0.42	11
Hippocampus	Imipramine	2.71 ± 0.31	10	2.39 ± 0.37	10
Hippocampus	AD20	2.88 ± 0.42	13	2.18 ± 0.24	13
Midbrain	DMSO	53.28 ± 8.88	11	40.01 ± 6.26	11
Midbrain	Imipramine	43.80 ± 7.07	10	36.78 ± 9.89	10
Midbrain	AD20	56.97 ± 12.07	13	40.74 ± 7.60	13

## Data Availability

The original contributions presented in this study are included in the article and the Supplementary Materials. Further inquiries can be directed to the corresponding author.

## Supplementary Materials

The following supporting information can be downloaded at https://www.mdpi.com/article/10.3390/cimb48090922/s1.

## References

[1] World Health Organization Depressive Disorder (Depression). Fact Sheet, 29 August 2025. https://www.who.int/news-room/fact-sheets/detail/depression.

[2] World Health Organization Over a Billion People Living with Mental Health Conditions—Services Require Urgent Scale-Up. News Release, 2 September 2025. https://www.who.int/news/item/02-09-2025-over-a-billion-people-living-with-mental-health-conditions-services-require-urgent-scale-up.

[3] McIntyre R.S., Alsuwaidan M., Baune B.T., Berk M., Demyttenaere K., Goldberg J.F., Gorwood P., Ho R., Kasper S., Kennedy S.H. (2023). Treatment-resistant depression: Definition, prevalence, detection, management, and investigational interventions. World Psychiatry.

[4] Kajumba M.M., Kakooza-Mwesige A., Nakasujja N., Koltai D., Canli T. (2024). Treatment-resistant depression: Molecular mechanisms and management. Mol. Biomed..

[5] Kositsyn Y.M., de Abreu M.S., Kolesnikova T.O., Lagunin A.A., Poroikov V.V., Harutyunyan H.S., Yenkoyan K.B., Kalueff A.V. (2023). Towards novel potential molecular targets for antidepressant and antipsychotic drugs. Int. J. Mol. Sci..

[6] Ferreira F.J.N., Carneiro A.S. (2025). AI-driven drug discovery: A comprehensive review. ACS Omega.

[7] Nivatya H.K., Singh A., Kumar N., Sonam, Sharma L., Singh V., Mishra R., Gaur N., Mishra A.K. (2025). Assessing molecular docking tools: Understanding drug discovery and design. Future J. Pharm. Sci..

[8] Li Y., Yi J., Li H., Li K., Kang F., Deng Y., Wu C., Fu X., Jiang D., Cao D. (2025). Decoding the limits of deep learning in molecular docking for drug discovery. Chem. Sci..

[9] Gendelev L., Taylor J., Myers-Turnbull D., Chen S., McCarroll M.N., Arkin M.R., Kokel D., Keiser M.J. (2024). Deep phenotypic profiling of neuroactive drugs in larval zebrafish. Nat. Commun..

[10] Chaoul V., Dib E.-Y., Bedran J., Khoury C., Shmoury O., Harb F., Soueid J. (2023). Assessing drug administration techniques in zebrafish models of neurological disease. Int. J. Mol. Sci..

[11] Chauhan A., Raviraj T., Sobti R.C., Dutta A., Shukla M.K., Korinek M. (2026). Zebrafish models for neurological disorders: A platform for natural product-based drug discovery. Front. Nat. Prod..

[12] Wang Y., Takai R., Yoshioka H., Shirabe K. (2006). Characterization and expression of serotonin transporter genes in zebrafish. Tohoku J. Exp. Med..

[13] Norton W.H.J., Folchert A., Bally-Cuif L. (2008). Comparative analysis of serotonin receptor HTR1A/HTR1B families and transporter slc6a4a/b gene expression in the zebrafish brain. J. Comp. Neurol..

[14] Severinsen K., Sinning S., Müller H.K., Wiborg O. (2008). Characterisation of the zebrafish serotonin transporter functionally links TM10 to the ligand binding site. J. Neurochem..

[15] de Abreu M.S., Giacomini A.C.V.V., Demin K.A., Petersen E.V., Kalueff A.V. (2021). Understanding how stress responses and stress-related behaviors have evolved in zebrafish and mammals. Neurobiol. Stress.

[16] Xie Y., Meijer A.H., Schaaf M.J.M. (2021). Modeling inflammation in zebrafish for the development of anti-inflammatory drugs. Front. Cell Dev. Biol..

[17] Percie du Sert N., Hurst V., Ahluwalia A., Alam S., Avey M.T., Baker M., Browne W.J., Clark A., Cuthill I.C., Dirnagl U. (2020). The ARRIVE guidelines 2.0: Updated guidelines for reporting animal research. PLoS Biol..

[18] Vassiliev P.M., Komelkova M.V., Perfilev M.A., Kochetkov A.N., Sarapultsev A.P., Fedorov S.A., Platkovskii P.O., Utepova I.A., Deev S.L., Sarapultsev G.P. (2025). Neural Network Modeling of the Multitarget Serotonergic Mechanism of Cellular Stress. J. Ural Med. Acad. Sci..

[19] Vassiliev P.M., Maltsev D.V., Spasov A.A., Perfilev M.A., Skripka M.O., Kochetkov A.N. (2023). Consensus Ensemble Multitarget Neural Network Model of Anxiolytic Activity of Chemical Compounds and Its Use for Multitarget Pharmacophore Design. Pharmaceuticals.

[20] Trott O., Olson A.J. (2010). AutoDock Vina: Improving the speed and accuracy of docking with a new scoring function, efficient optimization, and multithreading. J. Comput. Chem..

[21] Utepova I.A., Trestsova M.A., Chupakhin O.N., Charushin V.N., Rempel A.A. (2015). Aerobic oxidative C–H/C–H coupling of azaaromatics with indoles and pyrroles in the presence of TiO_2_ as a photocatalyst. Green Chem..

[22] Utepova I.A., Chupakhin O.N., Trestsova M.A., Musikhina A.A., Kucheryavaya D.A., Charushin V.N., Rempel A.A., Kozhevnikova N.S., Valeeva A.A., Mikhaleva A.I. (2016). Direct functionalization of the C–H bond in (hetero)arenes: Aerobic photoinduced oxidative coupling of azines with aromatic nucleophiles in the presence of a CdS/TiO_2_ photocatalyst. Russ. Chem. Bull..

[23] Eremeev A.V., Andrianov V.G., Piskunova I.P. (1978). Synthesis of furazano [3,4-b]pyrazine derivatives. Chem. Heterocycl. Compd..

[24] Elliott A.J., Guzik H., Soler J.R. (1982). On the reaction of 2-aminopyridines with α-halocarbonyl compounds. J. Heterocycl. Chem..

[25] OECD (2025). Test No. 236: Fish Embryo Acute Toxicity (FET) Test.

[26] Steele W.B., Kristofco L.A., Corrales J., Saari G.N., Haddad S.P., Gallagher E.P., Kavanagh T.J., Kostal J., Zimmerman J.B., Voutchkova-Kostal A. (2018). Comparative behavioral toxicology with two common larval fish models: Exploring relationships among modes of action and locomotor responses. Sci. Total Environ..

[27] Pohl J. (2019). Zebrafish (*Danio rerio*) embryo-larvae locomotor activity data analysis: Evaluating anxiolytic effects of the antidepressant compound citalopram. Data Brief.

[28] Goolish E.M., Okutake K. (1999). Lack of gas bladder inflation by the larvae of zebrafish in the absence of an air–water interface. J. Fish Biol..

[29] MacPhail R.C., Brooks J., Hunter D.L., Padnos B., Irons T.D., Padilla S. (2009). Locomotion in larval zebrafish: Influence of time of day, lighting and ethanol. Neurotoxicology.

[30] McCarroll M.N., Gendelev L., Kinser R., Taylor J., Bruni G., Myers-Turnbull D., Helsell C., Carbajal A., Rinaldi C., Kang H.J. (2019). Zebrafish behavioural profiling identifies GABA and serotonin receptor ligands related to sedation and paradoxical excitation. Nat. Commun..

[31] Zhdanov A.V., Khatsko S.L., Zabegalov K.N., Bytov M.V., Demin K.A., Galstyan D.S., de Abreu M.S., Amstislavskaya T.G., Kalueff A.V. (2025). Modeling stress-related disorders in zebrafish using prolonged predator exposure and prolonged unpredictable stress. J. Neurosci. Res..

[32] Naumenko V.S., Kondaurova E.M., Bazovkina D.V., Tsybko A.S., Ilchibaeva T.V., Khotskin N.V., Semenova A.A., Popova N.K. (2014). Effect of GDNF on depressive-like behavior, spatial learning and key genes of the brain dopamine system in genetically predisposed to behavioral disorders mouse strains. Behav. Brain Res..

[33] Tikhonova M.A., Lebedeva V.V., Kulikov A.V., Bazovkina D.V., Popova N.K. (2006). Effect of imipramine on the behavior and cerebral 5-HT1A serotonin receptors in mice genetically predisposed to catalepsy. Bull. Exp. Biol. Med..

[34] Kulikov A.V., Naumenko V.S., Voronova I.P., Tikhonova M.A., Popova N.K. (2005). Quantitative RT-PCR assay of 5-HT1A and 5-HT2A serotonin receptor mRNAs using genomic DNA as an external standard. J. Neurosci. Methods.

[35] Naumenko V.S., Kulikov A.V. (2006). Quantitative assay of 5-HT1A receptor gene expression in the brain. Mol. Biol..

[36] Naumenko V.S., Osipova D.V., Kostina E.V., Kulikov A.V. (2008). Utilization of a two-standard system in real-time PCR for quantification of gene expression in the brain. J. Neurosci. Methods.

[37] Devi N., Singh D., Rawal R.K., Bariwal J., Singh V. (2016). Medicinal attributes of imidazo [1,2-a]pyridine derivatives: An update. Curr. Top. Med. Chem..

[38] Xiong Y., Feichtinger K., Ren A.S., Ulmann B. (2013). Imidazo [1,2-a]pyridine Derivatives as Modulators of the 5-HT2A Serotonin Receptor Useful for the Treatment of Disorders Related Thereto.

[39] Tea M., Pan Y.K., Lister J.G.R., Perry S.F., Gilmour K.M. (2024). Effects of serta and sertb knockout on aggression in zebrafish (*Danio rerio*). J. Comp. Physiol. A Neuroethol. Sens. Neural Behav. Physiol..

[40] Connors K.A., Valenti T.W., Lawless K., Sackerman J., Onaivi E.S., Brooks B.W., Gould G.G. (2014). Similar anxiolytic effects of agonists targeting serotonin 5-HT1A or cannabinoid CB receptors on zebrafish behaviour in novel environments. Aquat. Toxicol..

[41] Sinning S., Musgaard M., Jensen M., Severinsen K., Celik L., Koldsø H., Meyer T., Bols M., Jensen H.H., Schiøtt B. (2010). Binding and orientation of tricyclic antidepressants within the central substrate site of the human serotonin transporter. J. Biol. Chem..

[42] Sarker S., Weissensteiner R., Steiner I., Sitte H.H., Ecker G.F., Freissmuth M., Sucic S. (2010). The high-affinity binding site for tricyclic antidepressants resides in the outer vestibule of the serotonin transporter. Mol. Pharmacol..

[43] Andersen J., Taboureau O., Hansen K.B., Olsen L., Egebjerg J., Strømgaard K., Kristensen A.S. (2009). Location of the antidepressant binding site in the serotonin transporter: Importance of Ser-438 in recognition of citalopram and tricyclic antidepressants. J. Biol. Chem..

[44] Zhou Z., Zhen J., Karpowich N.K., Law C.J., Reith M.E.A., Wang D.N. (2009). Antidepressant specificity of serotonin transporter suggested by three LeuT–SSRI structures. Nat. Struct. Mol. Biol..

