# Supplementary material for: Cross-Species Triage of SERT-Oriented Polyheteroaryl Candidates Prioritizes AD20/UtIA-0108 as an Early Neuroactive Candidate
Source: Curr Issues Mol Biol. 2026 Sep 9;48(9):922. doi: 10.3390/cimb48090922 (PMC13605482; doi:10.3390/cimb48090922)
Supplement: Supplementary file 1 [file cimb-48-00922-s001.zip › cimb-4524649-supplementary-english.pdf]

Electronic supplementary materials for the article

Cross-species triage of SERT-oriented polyheteroaryl candidates prioritizes AD20/UtIA-0108 as an early neuroactive candidate

Maria V. Komelkova [1], Stanislav A. Fedorov [1], Veronika A. Isaeva [2], Irina A. Utepova [1], Yulia D. Grygoreva [3], Inessa Yu Bahareva [3], Vitalii S. Moskaliuk [3], Elena M. Kondaurova [3], Alexandr V. Zhdanov [1], Pavel O. Platkovskii [1], Maria A. Trestsova [1,4,5], Daria A. Andreeva [4], Maxim A. Perfilev [6], Andrey N. Kochetkov [6], Pavel M. Vassiliev [1,6], Vladimir S. Naumenko [3] and Alexey P. Sarapultsev [1,*]

[1] Russian-Chinese Education and Research Center of System Pathology, South Ural State University, 76 Lenin Prospekt, 454080 Chelyabinsk, Russia; mkomelkova@mail.ru (M.V.K.); irina-utepova@yandex.ru (I.A.U.)

[2] Laboratory of Immunopathophysiology, Institute of Immunology and Physiology, Ural Branch of the Russian Academy of Sciences, 106 Pervomaiskaya Street, 620049 Ekaterinburg, Russia

[3] Federal Research Center Institute of Cytology and Genetics, Siberian Branch of the Russian Academy of Sciences, 10 Prospekt Akademika Lavrent'yeva, 630090 Novosibirsk, Russia

[4] Department of Organic and Biomolecular Chemistry, Ural Federal University, 28 Mira Street, 620002 Ekaterinburg, Russia

[5] Institute of Organic Synthesis, Ural Branch of the Russian Academy of Sciences, 22 S. Kovalevskoy Street, 620066 Ekaterinburg, Russia

[6] Scientific Center for Innovative Medicines, Volgograd State Medical University, 1 Pavshikh Bortsov Sq., 400066 Volgograd, Russia

* Correspondence: sarapultsevap@susu.ru

Contents

S1. Copies of NMR spectra of compounds **3a–c**
- Figure S1. ^{1}H NMR spectrum of compound **3a**
- Figure S2. ^{13}C NMR spectrum of compound **3a**
- Figure S3. ^{1}H NMR spectrum of compound **3b**
- Figure S4. ^{13}C NMR spectrum of compound **3b**
- Figure S5. ^{1}H NMR spectrum of compound **3c**
- Figure S6. ^{13}C NMR spectrum of compound **3c**

S2. Copies of MS and HRMS data for compounds **3a-c**
- Figure S7. MS spectrum of compound **3a**
- Figure S8. HRMS spectrum of compound **3a**
- Figure S9. MS spectrum of compound **3b**
- Figure S10. HRMS spectrum of compound **3b**
- Figure S11. MS spectrum of compound **3c**
- Figure S12. HRMS spectrum of compound **3c**

Table S1. qPCR primer sequences and characteristics

S3. Exploratory whole-body cortisol data

Table S2. Exploratory whole-body cortisol comparisons (chronic adult-zebrafish cohort)

S1. NMR spectra of compounds 3a–c.

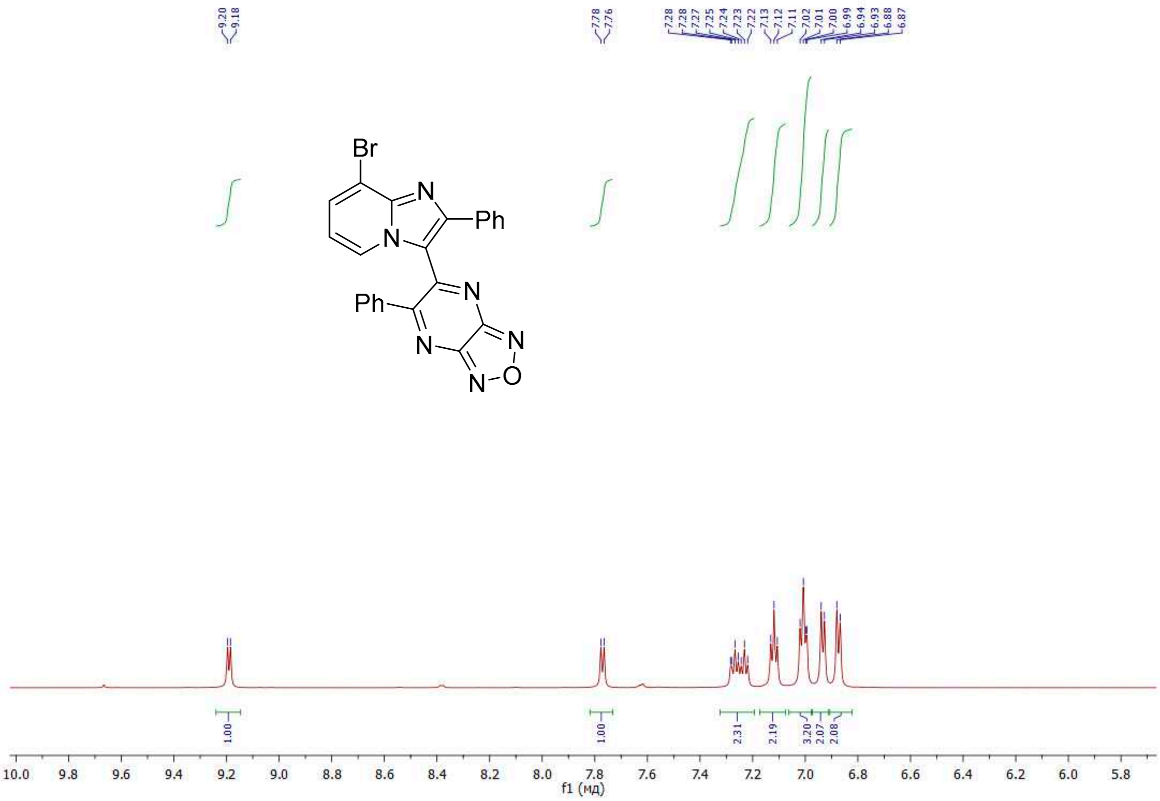

Figure S1. ^{1}H NMR Spectrum of 3a.

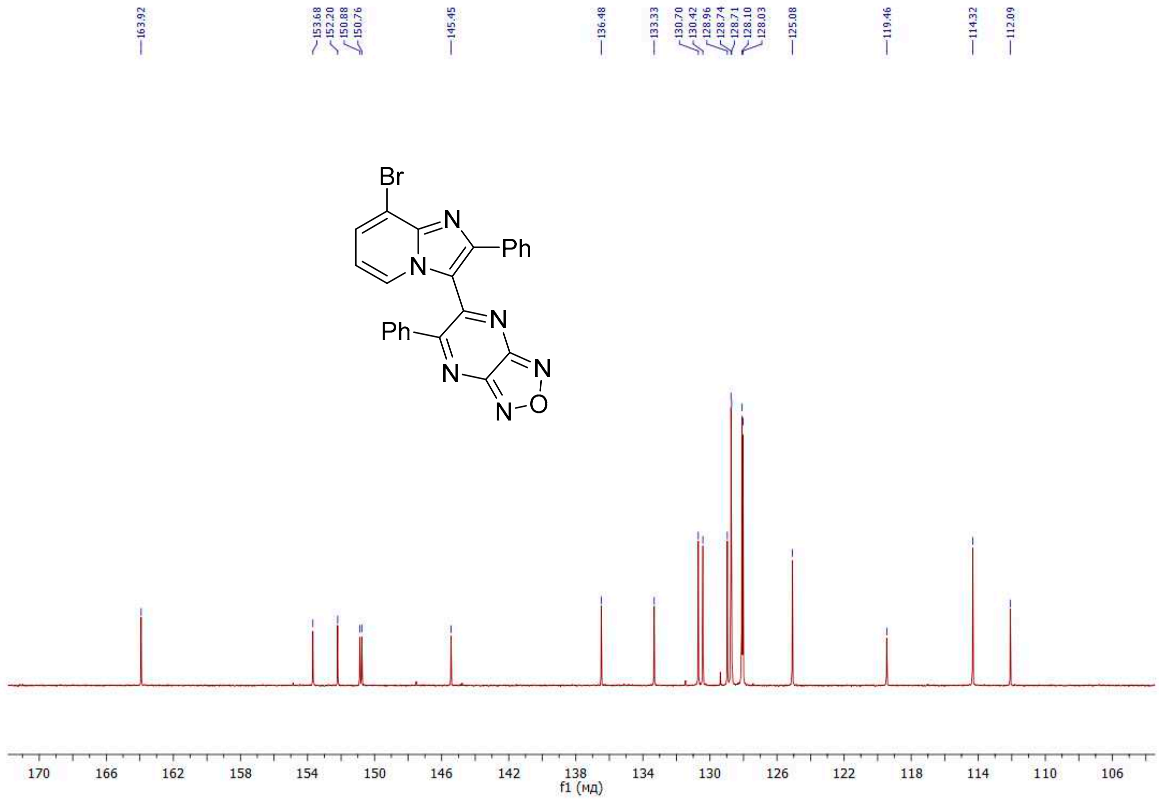

Figure S2. ^{13}C NMR Spectrum of 3a.

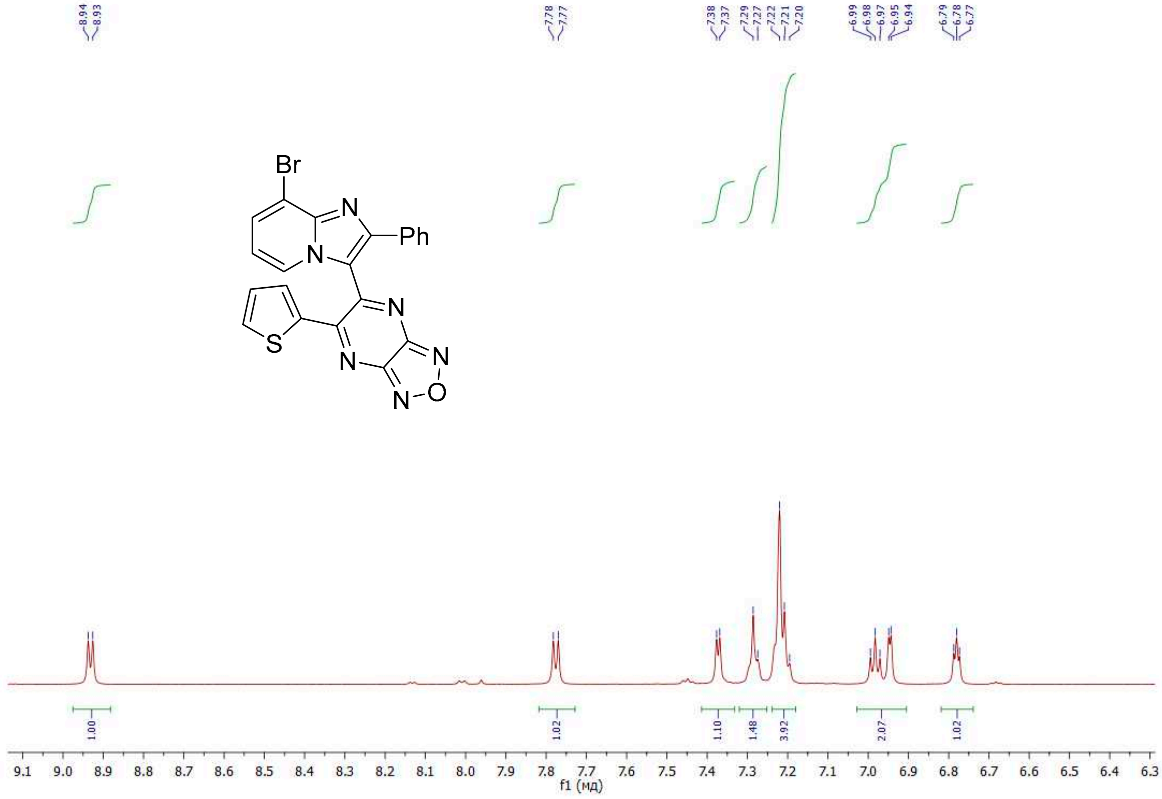

Figure S3. ^{1}H NMR Spectrum of 3b.

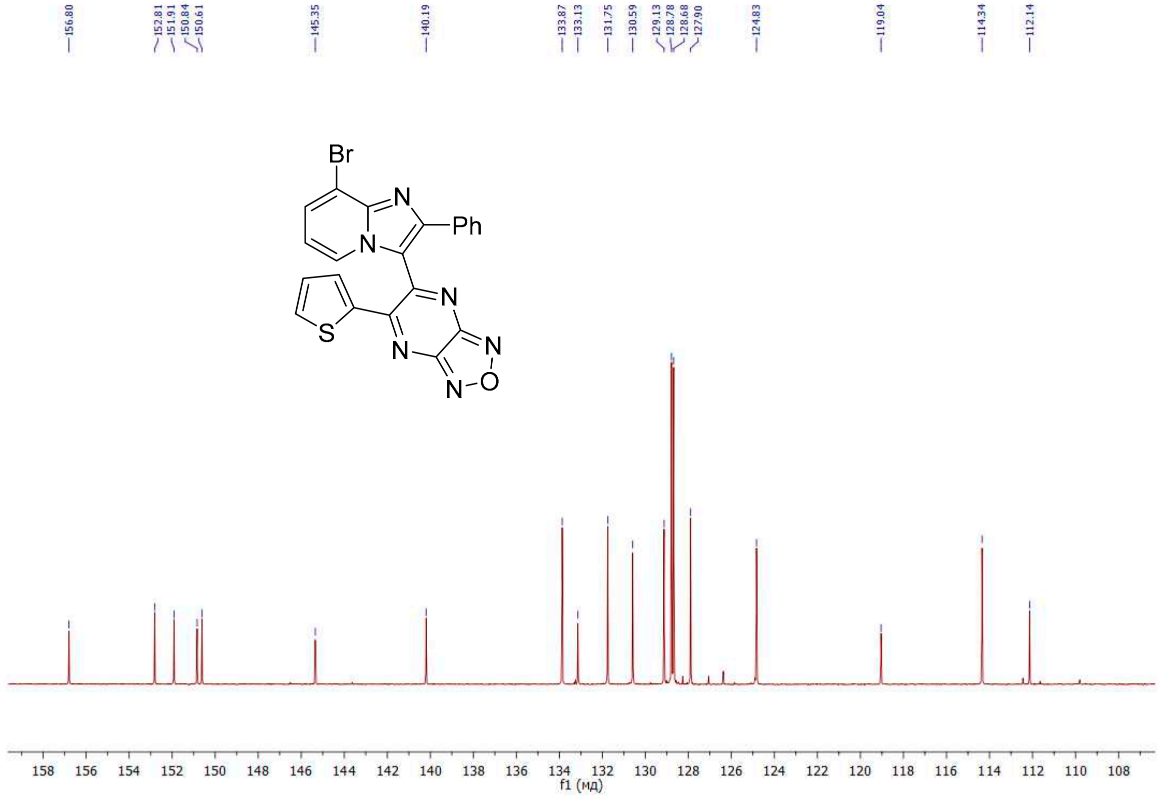

Figure S4. ^{13}C NMR Spectrum of 3b.

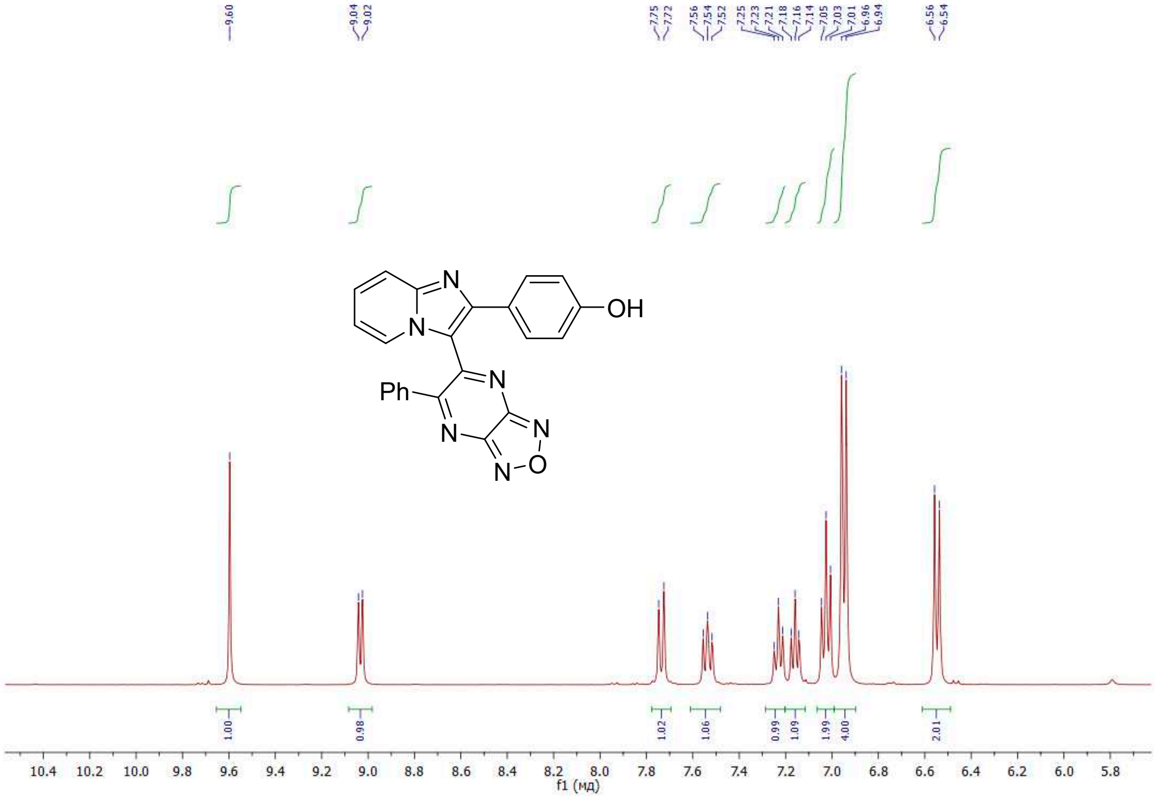

Figure S5. ^{1}H NMR Spectrum of 3c.

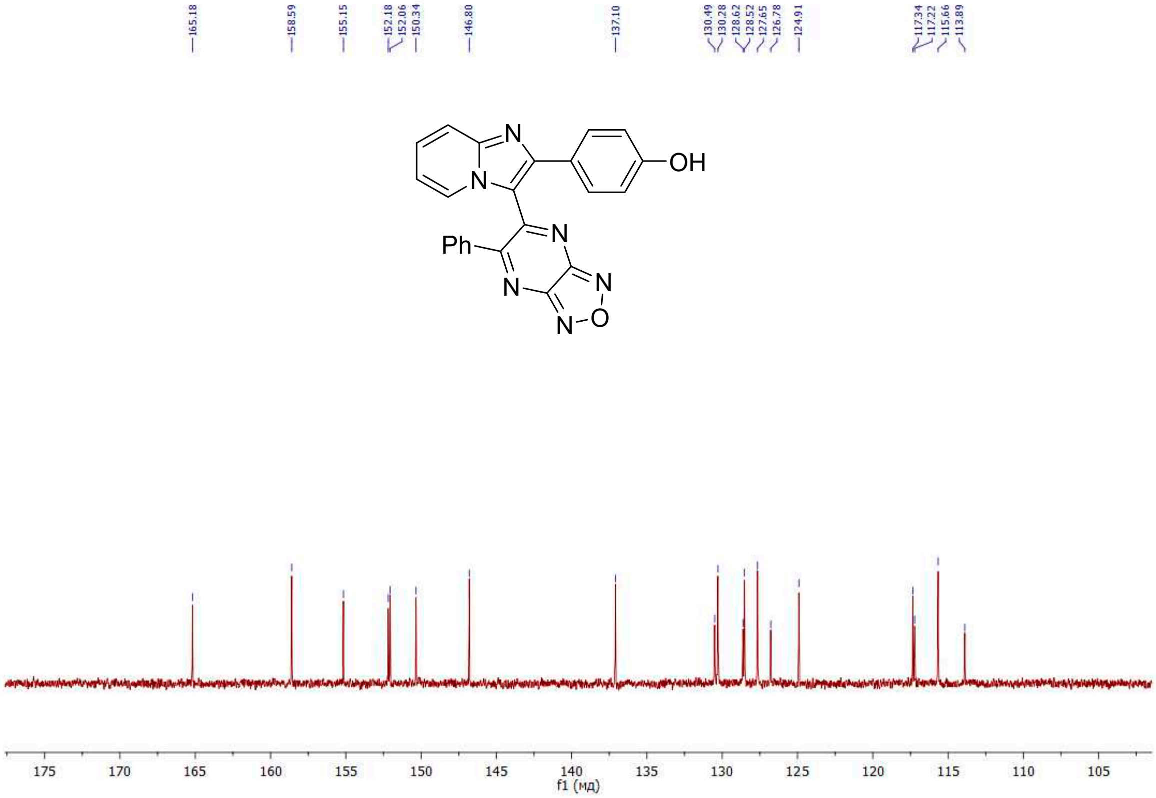

Figure S6. ^{13}C NMR Spectrum of 3c.

S2. Copies of MS and HRMS data for compounds 3a-c

Analyzed by	: Admin
Analyzed	: 11.05.2022 17:01:28
Sample Type	: Unknown
Level #	: 1
Sample Name	: AD19
Sample ID	: 4034
IS Amount	: [1]=1
Sample Amount	: 1
Dilution Factor	: 1
Vial #	: 1
Injection Volume	: 1.00
Modified by	: Admin
Modified	: 11.05.2022 17:04:52

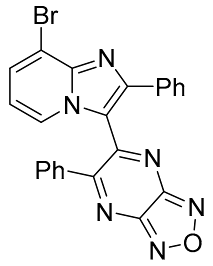

[Comment]

===== Analytical Line 1 =====

[GCMS-QP2010 Ultra]

IonSourceTemp	:200.00 °C
Interface Temp.	:150.00 °C
Solvent Cut Time	:0.00 min
Detector Gain Mode	:Relative to the Tuning Result
Detector Gain	:+0.00 kV
Threshold	:0

[MS Table]
--Group 1 - Event 1--

Start Time	:0.10min
End Time	:30.00min
ACQ Mode	:Scan
Event Time	:0.15sec
Scan Speed	:5000
Start m/z	:35.00
End m/z	:550.00

Sample Inlet Unit :DI

[DI Temperature Program]

Rate (°C/min)	Final Temp.(°C)	Hold Time
80	200	0.00
80	250	0.00
80	300	1.00
80	350	1.00
80	440	5.00

Line#:1 R.Time:2.860(Scan#:1105)
MassPeaks:276
RawMode:Single 2.860(1105) BasePeak:470(2430278)
BG Mode:3.038(1176) Group 1 - Event 1

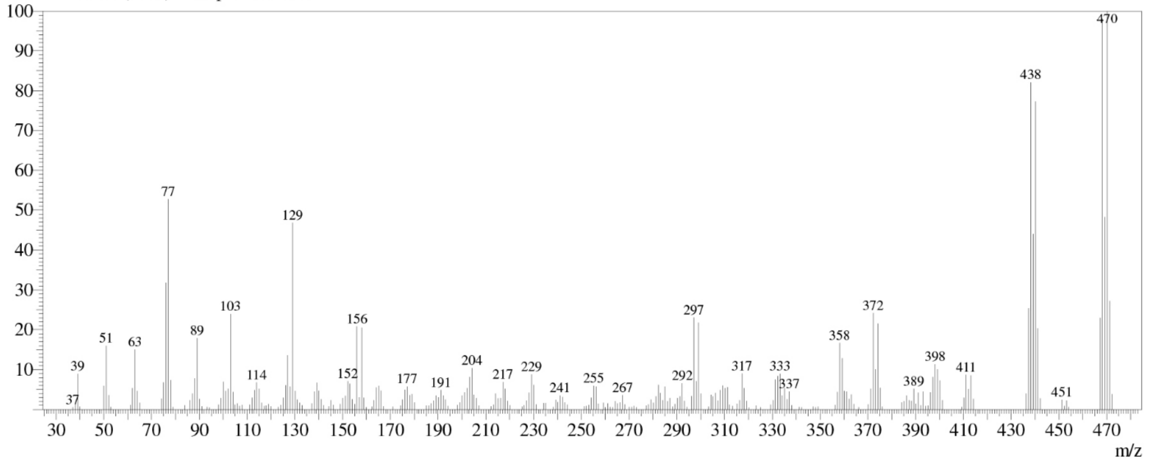

Figure S7. MS spectrum of compound 3a.

Analysis Info

Analysis Name	D:\Data\ING26\ZE-AD-19.02D-B.9625_EN180_02D1430.d	Acquisition Date	4/2/2026 2:31:08 PM
Method	DLIEN180YA27_HPC50-2300_EPO100C4500N0.2DG4.0DT200_f1250f2350hrf60ie8lm50ce8pps5crf300-1000tt70-130_St_F_3x1_Segm1.m	Operator	ing@ios.uran.ru
Sample Name		Instrument	maXis impact
Comment	Initial calibration: HPC (HCOONa)<br>Background: -364.05 (#9612), -349.03 (#9618); 513.02/515.02 (#9622)		

Acquisition Parameter

Source Type	ESI	Ion Polarity	Negative	Set Nebulizer	0.2 Bar
Focus	Active	Set Capillary	4500 V	Set Dry Heater	200 °C
Scan Begin	50 m/z	Set End Plate Offset	-100 V	Set Dry Gas	4.0 l/min
Scan End	3300 m/z	Set Charging Voltage	2000 V	Set Divert Valve	Source
		Set Corona	0 nA	Set APCI Heater	0 °C

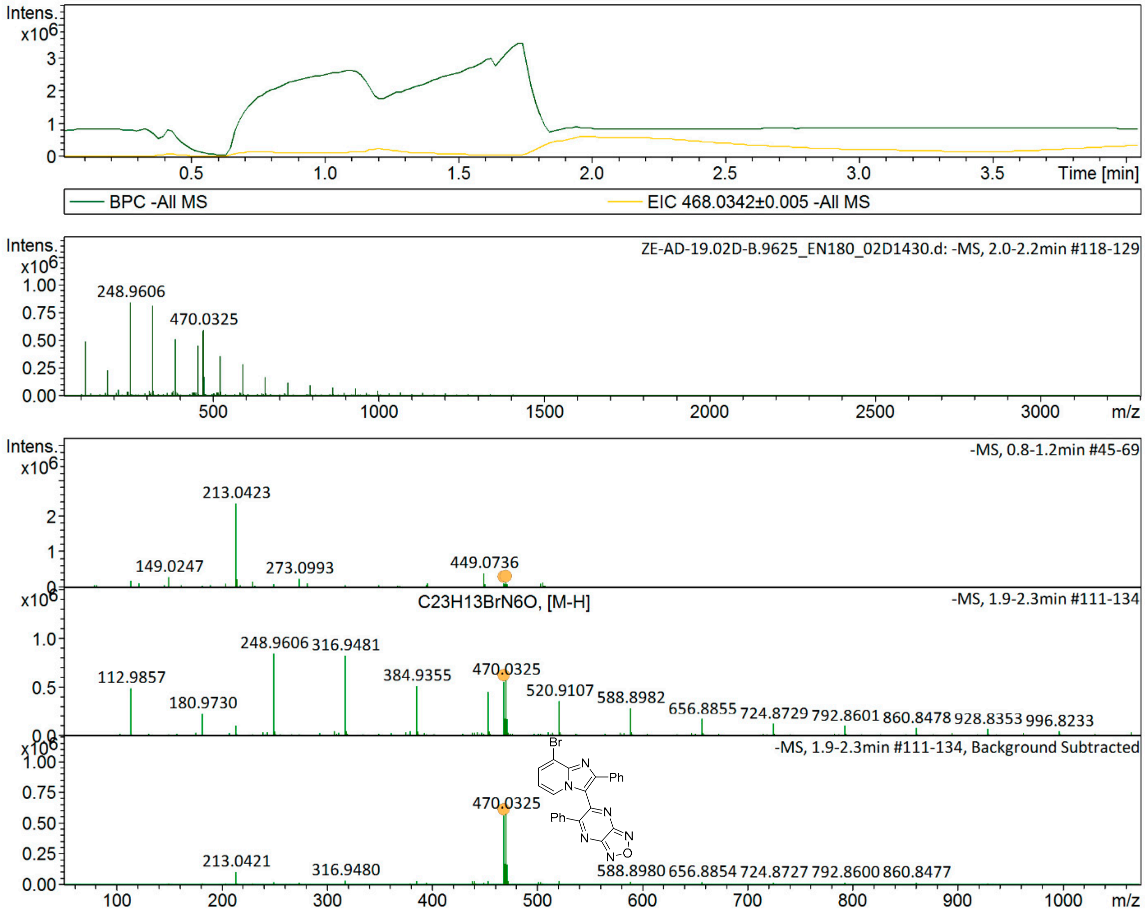

Figure S8. HRMS spectrum of compound 3a.

Analyzed by	: Admin
Analyzed	: 01.03.2022 16:26:12
Sample Type	: Unknown
Level #	: 1
Sample Name	: AD20
Sample ID	: 3658
IS Amount	: [1]=1
Sample Amount	: 1
Dilution Factor	: 1
Vial #	: 1
Injection Volume	: 1.00
Modified by	: Admin
Modified	: 01.03.2022 16:29:25

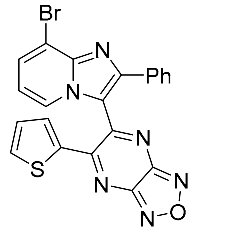

Molecular Weight: 475,32

[Comment]

===== Analytical Line 1 =====

[GCMS-QP2010 Ultra]

IonSourceTemp	:200.00 °C
Interface Temp.	:150.00 °C
Solvent Cut Time	:0.00 min
Detector Gain Mode	:Relative to the Tuning Result
Detector Gain	:+0.00 kV
Threshold	:0

[MS Table]
--Group 1 - Event 1--

Start Time	:0.10min
End Time	:30.00min
ACQ Mode	:Scan
Event Time	:0.15sec
Scan Speed	:5000
Start m/z	:35.00
End m/z	:550.00
Sample Inlet Unit	:DI

[DI Temperature Program]

Rate (°C/min)	Final Temp.(°C)	Hold Time
80	200	0.00
80	250	0.00
80	300	1.00
80	350	1.00
80	440	5.00

Line#:1 R.Time:2.760(Scan#:1065)
MassPeaks:354
RawMode:Single 2.760(1065) BasePeak:476(1096997)
BG Mode:1.395(519) Group 1 - Event 1

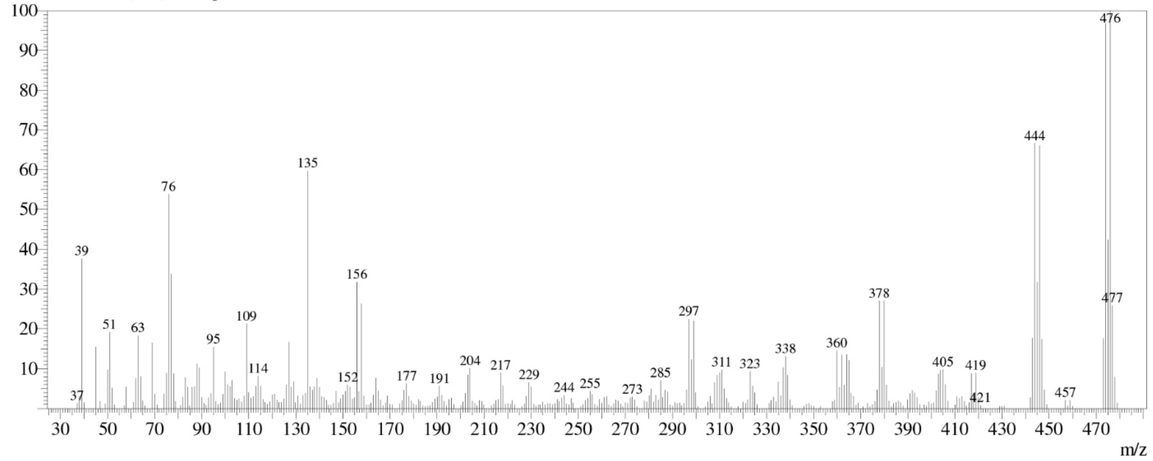

Figure S9. MS spectrum of compound 3b.

Analysis Info

Analysis Name	D:\Data\ING26\ZE-AD-20.02D-B.9624_EN180_02D1415.d	Acquisition Date	4/2/2026 2:12:48 PM
Method	DLIEN180YA27_HPC50-2300_EPO100C4500N0.2DG4.0DT200_f1250f2350hrf60ie8lm50ce8pps5crf300-1000tt70-130_St_F_3x1_Segm1.m	Operator	ing@ios.uran.ru
Sample Name		Instrument	maXis impact
Comment	Initial calibration: HPC (HCOONa)<br>Background: -364.05 (#9612), -349.03 (#9618); 513.02/515.02 (#9622)		

Acquisition Parameter

Source Type	ESI	Ion Polarity	Negative	Set Nebulizer	0.2 Bar
Focus	Active	Set Capillary	4500 V	Set Dry Heater	200 °C
Scan Begin	50 m/z	Set End Plate Offset	-100 V	Set Dry Gas	4.0 l/min
Scan End	3300 m/z	Set Charging Voltage	2000 V	Set Divert Valve	Source
		Set Corona	0 nA	Set APCI Heater	0 °C

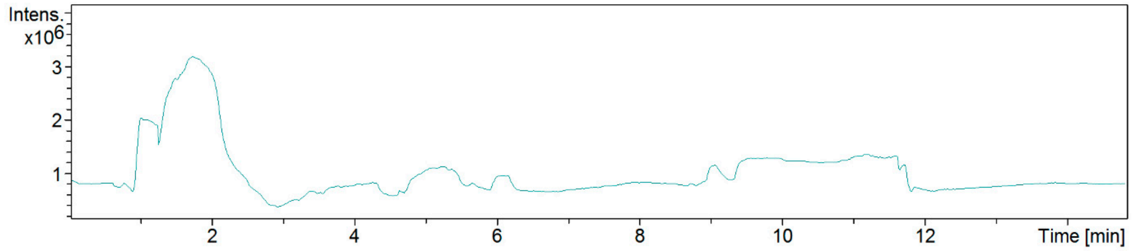

-MS, 4.1-7.7min #245-458

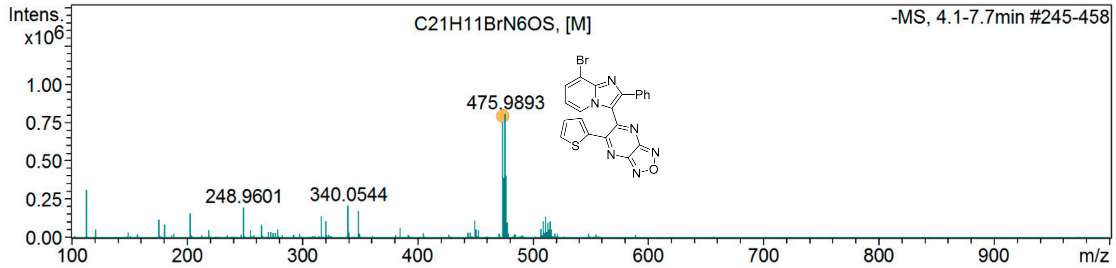

Figure S10. HRMS spectrum of compound 3b.

Analyzed by	: Admin
Analyzed	: 02.11.2024 12:34:13
Sample Type	: Unknown
Level #	: 1
Sample Name	: AD12
Sample ID	: 1017
IS Amount	: [1]=1
Sample Amount	: 1
Dilution Factor	: 1
Vial #	: 1
Injection Volume	: 1.00
Modified by	: Admin
Modified	: 02.11.2024 12:39:54

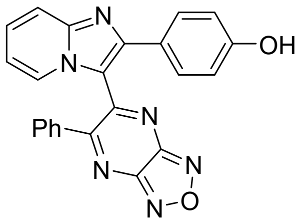

[Comment]

===== Analytical Line 1 =====

[GCMS-QP2010 Ultra]

IonSourceTemp	:200.00 °C
Interface Temp.	:250.00 °C
Solvent Cut Time	:0.00 min
Detector Gain Mode	:Relative to the Tuning Result
Detector Gain	:+0.00 kV
Threshold	:0

[MS Table]
--Group 1 - Event 1--

Start Time	:0.10min
End Time	:30.00min
ACQ Mode	:Scan
Event Time	:0.15sec
Scan Speed	:5000
Start m/z	:35.00
End m/z	:750.00
Sample Inlet Unit	:DI

[DI Temperature Program]

Rate (°C/min)	Final Temp.(°C)	Hold Time
80	200	0.00
80	250	0.00
80	300	1.00
80	350	1.00
80	440	5.00

Line#:1 R.Time:3.407(Scan#:1324)
MassPeaks:198
RawMode:Single 3.408(1324) BasePeak:406(3253945)
BG Mode:2.210(845) Group 1 - Event 1

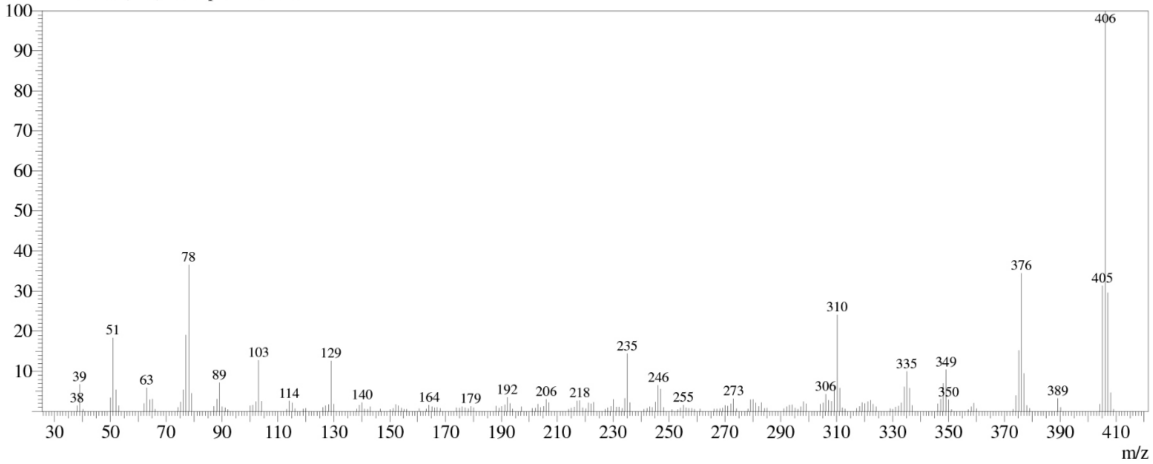

Figure S11. MS spectrum of compound 3c.

Analysis Info

Analysis Name	D:\Data\ING26\ZE-AD12.25C-B.9580_EN180_25C1425.d	Acquisition Date	3/25/2026 2:26:56 PM
Method	DLIEN180YA27_HPC50-2300_EPO100C4500N0.2DG4.0DT200_f1250f2350hrf60ie8lm50ce8pps5crf300-1000tt70-130_St_F_3x1_Segm1.m	Operator	ing@ios.uran.ru
Sample Name		Instrument	maXis impact
Comment	HCOONa		

Acquisition Parameter

Source Type	ESI	Ion Polarity	Negative	Set Nebulizer	0.2 Bar
Focus	Active	Set Capillary	4500 V	Set Dry Heater	200 °C
Scan Begin	50 m/z	Set End Plate Offset	-100 V	Set Dry Gas	4.0 l/min
Scan End	3300 m/z	Set Charging Voltage	2000 V	Set Divert Valve	Source
		Set Corona	0 nA	Set APCI Heater	0 °C

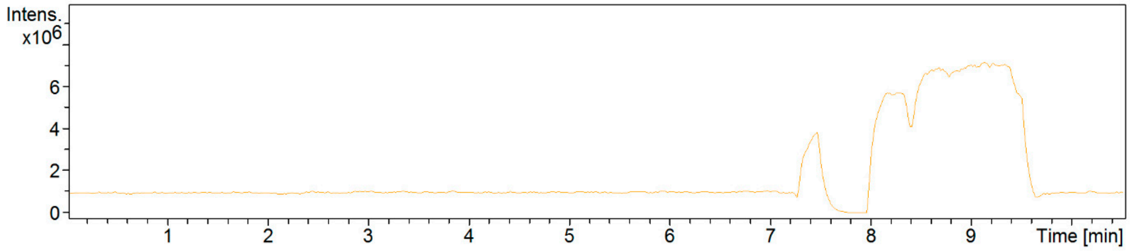

-MS, 8.7-8.9min #521-529

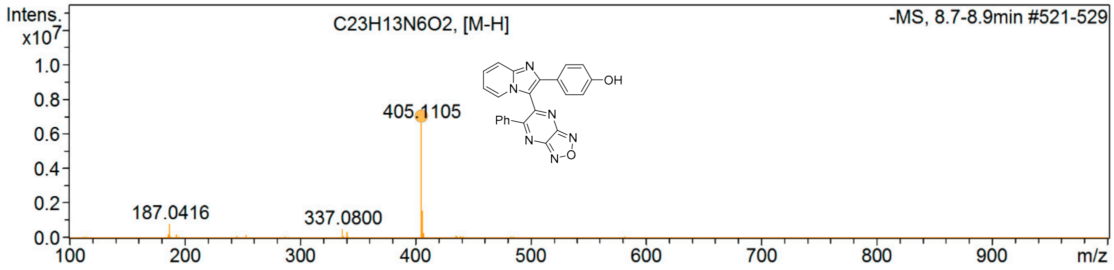

Figure S12. HRMS spectrum of compound 3c.

Table S1. Full qPCR primer panel (mouse). Genes used in the main Results and Figure 6 are listed in the main text (Table 3); Htr1a and Htr7 were assayed but are not analyzed in the main text.

Gene	Forward primer (5′→3′)	Reverse primer (5′→3′)	T_{ann} (°C)	Product (bp)
Bdnf	tacctggatgccgcaaacat	tgcttcagttggcctttgga	60	191
Creb1	gctggctaacaatggtacggat	tggttgctgggcactagaat	64	140
Maoa	aatgaggatgttaaatgggtagatgttggt	cttgacatattcaactagacgctc	63	138
Slc6a4	cgctctactacctcatctcctcc	gtcctgggcgaagtagttgg	63	101
Tph2	cattcctcgcacaattccagtcg	agtctacatccatcccaactgctg	61	239
Htr1a	ctgtgacctgtttatcgccctg	gtagtctatagggtcggtgattgc	62	109
Htr7	ggctacacgatctactccaccg	cgcacactcttccacctccttc	65	198
Polr2a (reference)	tgtgacaactccatacaatgc	ctctcttagtgaatttgcgtact	60	194

S3. Exploratory whole-body cortisol data

These whole-body cortisol measurements from the chronic adult zebrafish cohort are retained in the Supplementary Materials for transparency and are not used in the revised manuscript as evidence supporting AD20 activity or validation of the CUS model. Complete assay-platform and tissue-extraction details could not be recovered from the retained records; therefore, the comparisons are considered exploratory. After Holm correction, only the CUS+imipramine versus CUS comparison remained significant; whereas, CUS+AD20 versus CUS was not significant.

Table S2. Exploratory whole-body cortisol comparisons (chronic adult-zebrafish cohort). Whole-body cortisol (ng/g tissue). Holm correction was applied across the family of seven pairwise tests. Nominal (uncorrected) values are reported for transparency only.

Comparison	n (group 1)	Mean 1 (ng/g)	n (group 2)	Mean 2 (ng/g)	Nominal p	Holm p	Status
CUS vs control	8	31.90	11	60.32	0.0203	0.1017	nominal only; ns after Holm
CUS+AD20 vs CUS	10	41.83	8	31.90	0.1220	0.2440	ns after Holm
CUS+AD19 vs CUS	9	57.19	8	31.90	0.0111	0.0666	nominal only; ns after Holm
CUS+imipramine vs CUS	9	62.25	8	31.90	0.00156	0.0109	significant after Holm
control+AD20 vs control	9	55.15	11	60.32	0.704	0.758	ns after Holm
control+AD19 vs control	10	45.50	11	60.32	0.379	0.758	ns after Holm
control+imipramine vs control	12	43.32	11	60.32	0.186	0.557	ns after Holm
